# Targeting the interplay of cGAS-STING and ferroptosis by nanomedicine in the treatment of cancer

**DOI:** 10.1186/s13046-025-03520-6

**Published:** 2025-08-22

**Authors:** Chunfei Li, Wenyan Zhao, Donghua Geng, Yuzi Jin, Wenzheng Guan

**Affiliations:** 1 Department of General Surgery, Shengjing Hospital of China Medical University, Shenyang, 110004 China; 2 Department of Pediatrics, Central Hospital Affiliated to Shenyang Medical College, Shenyang, 110020 China; 3 Center of Reproductive Medicine, Shengjing Hospital of China Medical University, Shenyang, 110004 China

**Keywords:** Cancer, cGAS-STING, Ferroptosis, Nanomedicine

## Abstract

The cyclic GMP–AMP synthase (cGAS)–stimulator of interferon genes (STING) pathway senses cytosolic DNA and triggers innate immune responses. Pharmacological activation of the cGAS-STING pathway by cGAS-STING agonists to overcome cancer drug resistance offers substantial potential to promote antitumor immunity. However, small-molecule STING agonists show rapid excretion, low bioavailability, non-specificity, and adverse effects, which limit their therapeutic efficacy and in vivo applications. The recent emergence of nanomedicine has profoundly revolutionized STING agonist delivery, promoting tumor-targeted delivery and offering new opportunities for tumor-specific immunotherapy. A growing body of evidence has shown that cGAS-STING interacts with ferroptosis in cancer cells. Targeting the interplay between cGAS-STING and ferroptosis using nanomedicines offers a novel cancer treatment regimen. In this review, we outline the principal components of the cGAS-STING signaling cascade and discuss its role in cancer biology. We also review the role of the interplay between cGAS-STING and ferroptosis in cancer genesis. We then focus on providing an overview of the latest findings and emerging concepts that leverage the interplay between cGAS-STING and ferroptosis by nanomedicine to kill cancers. Finally, we discuss the key limitations of the current therapeutic paradigm and possible strategies to overcome them. This article highlights some promising therapeutic avenues that leverage the interplay of cGAS-STING and ferroptosis by nanomedicine, which could be used to treat cancer.

**Supplementary Information:**

The online version contains supplementary material available at 10.1186/s13046-025-03520-6.

## Introduction

Cancer metastasis refers to the growth and colonization of cancer cells in organs distant from the organ of origin. Metastasis, which characterizes a malignant tumor and distinguishes it from a benign tumor, is a hallmark of cancer associated with invasiveness [1, 2]. Metastasis is the ultimate and most lethal manifestation of cancer, leading to the vast majority (90%) of cancer deaths [3–6]. Immunotherapeutic agents such as anti-programmed death-ligand 1 (PD-L1) and anti-cytotoxic T lymphocyte -associated protein 4 (CTLA-4) inhibitors have emerged as revolutionary cancer treatments [7]. However, their low response rates, resistance, and toxicity pose substantial challenges. Blocking the programmed cell death protein 1 (PD-1)/PD-L1 pathway alone often fails to generate effective antitumor immune responses. Thus, developing rational drug combinations based on a deeper understanding of the molecular mechanisms underlying therapeutic resistance can help enhance treatment efficacy [8].

The cyclic GMP–AMP synthase (cGAS)–stimulator of interferon genes (STING) axis serves as a critical regulator of tissue homeostasis and the innate immune defense. Dysfunction of this pathway aberrantly activates pro-inflammatory cascades, contributing to the pathogenesis of inflammatory disorders, autoimmune conditions, degenerative diseases, and carcinogenesis [9–14]. In oncology research, cGAS -STING activation has been shown to contribute to antitumor effects by triggering spontaneous antitumor immunity, enhancing senescence in premalignant cells, facilitating conventional cancer therapy responses, and inducing regulated cell death through interferon (IFN)-dependent and IFN-independent pathways [15, 16]. Thus, this tumor-suppressor pathway can serve as a therapeutic target. Although recent advances in the development of cGAS-STING activators have contributed to tumor immunotherapy [17], the limitations of cGAS-STING activators, including drug resistance, low stability, poor half-life, off-target effects, and toxicity, have hampered their clinical application and greatly reduced their therapeutic efficacy. Because of these limitations, nonspecific delivery of cGAS-STING activators leads to unexpected immunotoxicities [18]. However, the emergence of nanomedicine has profoundly revolutionized STING agonist delivery, promoting tumor-targeted delivery and offering new opportunities for tumor-specific immunotherapy [19–24].

A growing body of evidence has shown that cGAS-STING interacts with regulated cell death (RCD), resulting in ferroptosis in cancers [2, 25–30]. Ferroptosis, a form of RCD induced by iron, is triggered by toxic accumulation of lipid peroxides on cellular membranes [31]. Conventional therapeutic modalities, including chemotherapy, immunotherapy, targeted cancer therapies, and radiotherapy, mediate tumor killing by inducing ferroptosis [31–35]. Therefore, ferroptosis offers great potential in cancer therapy, and targeting ferroptosis may provide new therapeutic opportunities for cancer treatment. Emerging evidence has shown that targeting the interplay between cGAS-STING and ferroptosis using nanomedicine represents a novel therapeutic regimen for cancers [36–40].

In this review, we first outline the principal components of the cGAS-STING signaling cascade and discuss its role in cancer biology. Then, we review the role of the interplay between cGAS-STING and ferroptosis in cancer genesis. We then focus on providing an overview of the latest findings and emerging concepts that leverage the interplay between cGAS-STING and ferroptosis by nanomedicine to kill cancers. Finally, we discuss the key limitations of the current therapeutic paradigm and propose possible strategies to overcome them. This article highlights some promising therapeutic avenues that leverage the interplay between cGAS-STING and ferroptosis using nanomedicine, which could be used to treat cancer.

## cGAS-STING signaling pathway

### Overview of the cGAS-STING signaling pathway

**Fig. 1:**
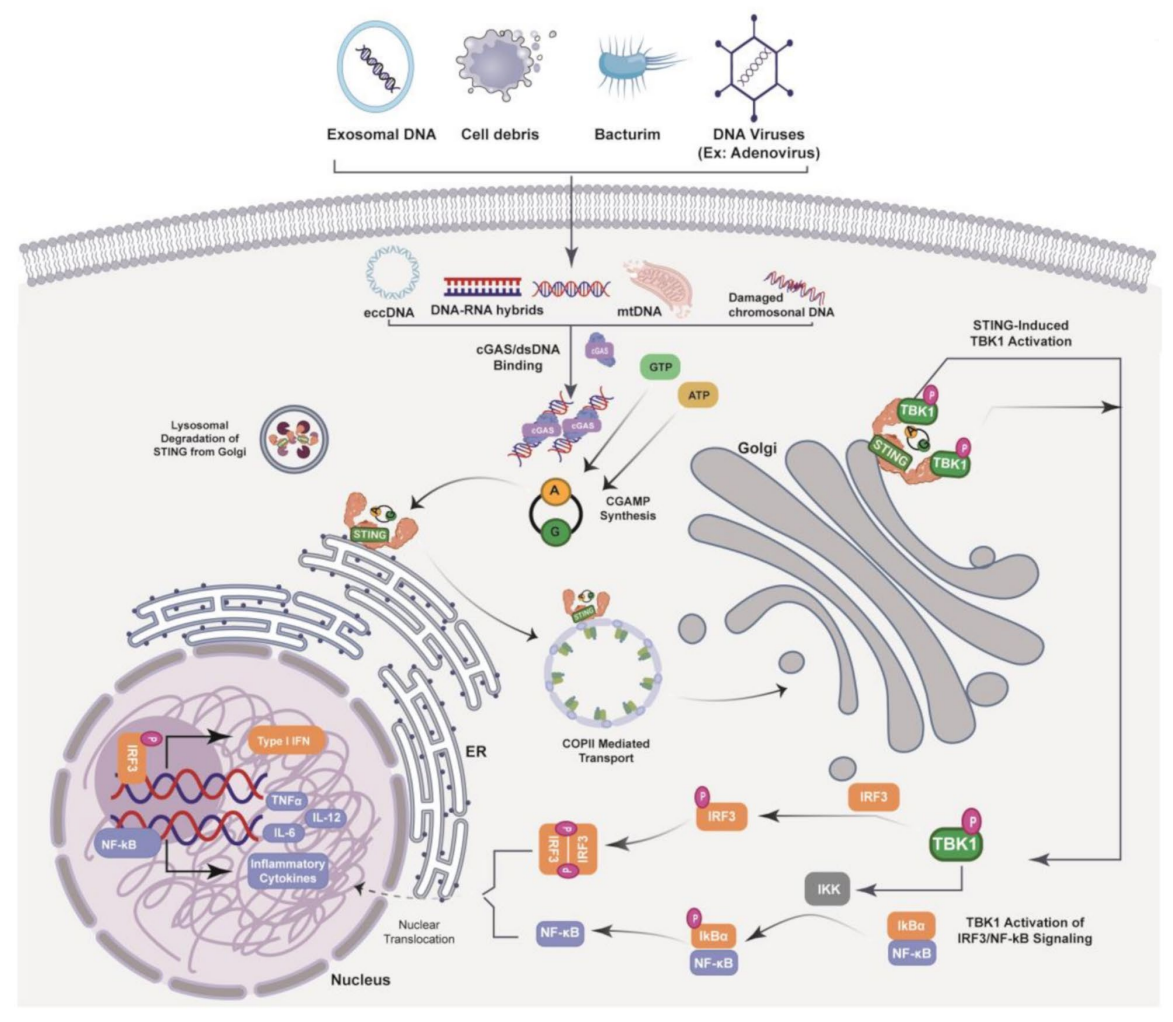
The cGAS-STING Signaling Cascade. The cGAS-STING pathway is initiated by the accumulation of cytosol double-stranded DNA(dsDNA), which are introduced by virus, bacteria, dead cells, mitochondria and cancer cells, et al. cGAS recognizes dsDNA exposed during pathogen infection or cellular stress, leading to produce the second messenger, 2′3′ cyclic GMP-AMP (cGAMP). Upon accumulation, cGAMP binds to STING located on the endoplasmic reticulum (ER) membrane and facilitates further pathway activation. The binding of cGAMP to STING mediates STING dimerization and translocation from the ER to the Golgi apparatus, where STING forms a complex with TANK-binding kinase 1 (TBK1), which, through auto-phosphorylation and STING phosphorylation, mediates recruitment

The cGAS-STING signaling axis consists of the synthase for the second messenger cyclic GMP–AMP (cGAS) and the cyclic GMP–AMP receptor STING (Fig. 1). cGAS is located upstream of STING [41, 42]. The cGAS-STING signaling axis detects pathogenic extranuclear DNA and triggers a type I interferon (IFN) innate immune response, which is physiologically used against microbial infections, making cGAS-STING a crucial component of the innate immune system [43]. cGAS senses microbial (viral, bacterial, and protozoan) double-stranded DNA (dsDNA) in a sequence-independent manner. It can be activated by endogenous DNA, mitochondria-released DNA, and genotoxic stress-mediated extranuclear chromatin, making cGAS-STING an important axis in autoimmunity, sterile inflammatory responses, and cellular senescence [43]. In mammalian cells, cGAS, the secondary messenger cyclic GMP–AMP (cGAMP) produced by cGAS, and cGAMP bound to STING form a crucial cytosolic DNA-sensing mechanism. Upon binding of dsDNA to cGAS, a conformational change occurs, activating it and initiating its enzymatic activity [44–48]. Active cGAS catalyzes and converts guanosine triphosphate (GTP) and adenosine triphosphate (ATP) into 2′,3′-cyclic GMP–AMP (cGAMP) [42]. Subsequently, 2ʹ3ʹ-cGAMP binds to and activates STING, a ~ 40-kDa endoplasmic reticulum (ER)-localized transmembrane protein adapter [42, 49, 50], to form dimers, tetramers, and higher-order oligomers [51, 52]. After activation, STING translocates from the ER to ER-Golgi intermediate compartments, where STING recruits TANK binding kinase 1 (TBK1) and IκB kinase (IKK), which respectively phosphorylate and activate interferon regulatory factor 3 (IRF3) and the nuclear factor (NF)-κB inhibitor IκBα [43]. TBK1 trans-phosphorylates itself, the C-terminal domain of STING, and subsequently IRF3 [43]. Meanwhile, STING engages with and activates IKK to trigger NF-κB signaling [43], which works together with a robust IFN response to orchestrate inflammatory immune responses to eliminate pathogens such as intracellular bacteria, retroviruses, and DNA viruses [43]. IRF3 dimerizes and translocates to the nucleus to transcriptionally activate genes encoding type I interferons such as IFN-β that together orchestrate antiviral defense mechanisms [43]. The phosphorylation of IκBα leads to translocation of NF-κB to the nucleus to enhance the transcription of genes encoding pro-inflammatory cytokines such as tumor necrosis factor (TNF) and IL-6 [53]. After activation, STING is trafficked to endolysosomes for degradation [43]. cGAS senses cytosolic dsDNA in response to tissue injury or pathogenic invasion, which allows the cGAS-STING axis to regulate various cellular functions such as protein synthesis, IFN/cytokine production, autophagy, senescence, metabolism, and specific mechanisms of cell death [43]. The cGAS-STING axis is vital for tissue homeostasis and host defense, and cGAS-STING dysfunction activates pro-inflammatory signaling pathways, resulting in inflammatory, autoimmune, and degenerative diseases and cancer [43].

### cGAS-STING in cancer

The epigenetic silencing of cGAS-STING represents a pervasive mechanism of immune evasion in cancer. Suppression of cGAS-STING has been reported in various human malignancies [54, 55]. The mechanisms underlying this suppression include epigenetic modifications, posttranslational modifications (PTMs), and various intracellular metabolic pathways that contribute significantly to therapeutic resistance. Enzymes that regulate these processes, such as DNA/histone modifiers, non-coding RNAs (ncRNAs), and 5-methylcytosine (m^5^C) modification writers, have also been identified. Abnormal epigenetic modifications contribute to disease initiation and progression by disrupting gene expression, altering protein profiles, and promoting the development of malignant phenotypes [56]. Recent studies have revealed that DNA methylation [57], histone methylation/demethylation [58–63], m^5^C modification [64], and non-coding RNA (ncRNA)-mediated epigenetic modifications [65, 66] induce the suppression of cGAS and STING in cancer. Emerging evidence has demonstrated that PTMs, including phosphorylation [67–69], ubiquitination [70–73], palmitoylation [74], methylation [75, 76], and lactylation [77], critically modulate the expression of cGAS and STING in cancer through multiple mechanisms. Emerging studies have also elucidated the metabolic pathways regulating cGAS and STING in cancer, including serine [78], fatty acid [79], glucose [64], purine synthesis [80], and ATP metabolism [81]. A better understanding of metabolic reprogramming mechanisms can reveal vulnerabilities that can be exploited to reactivate cGAS-STING signaling and enhance antitumor immunity.

In oncology, cGAS-STING activation contributes to antitumor effects by triggering spontaneous antitumor immunity, enhancing senescence in premalignant cells, facilitating conventional cancer therapy responses, and inducing RCD through IFN-dependent and IFN-independent pathways [15, 16, 82], establishing it as a tumor-suppressor pathway that can be therapeutically targeted. However, monotherapy with STING agonists has shown limited clinical efficacy and poor outcomes in patients with advanced cancer. Recent preclinical research, including in vitro and in vivo studies, have expanded our understanding of the molecular mechanisms by which cancer cells autonomously and non-autonomously evade cGAS-STING activation [57, 58, 60, 64, 65].

## Ferroptosis in cancers

### Core mechanism of ferroptosis

**Fig. 2:**
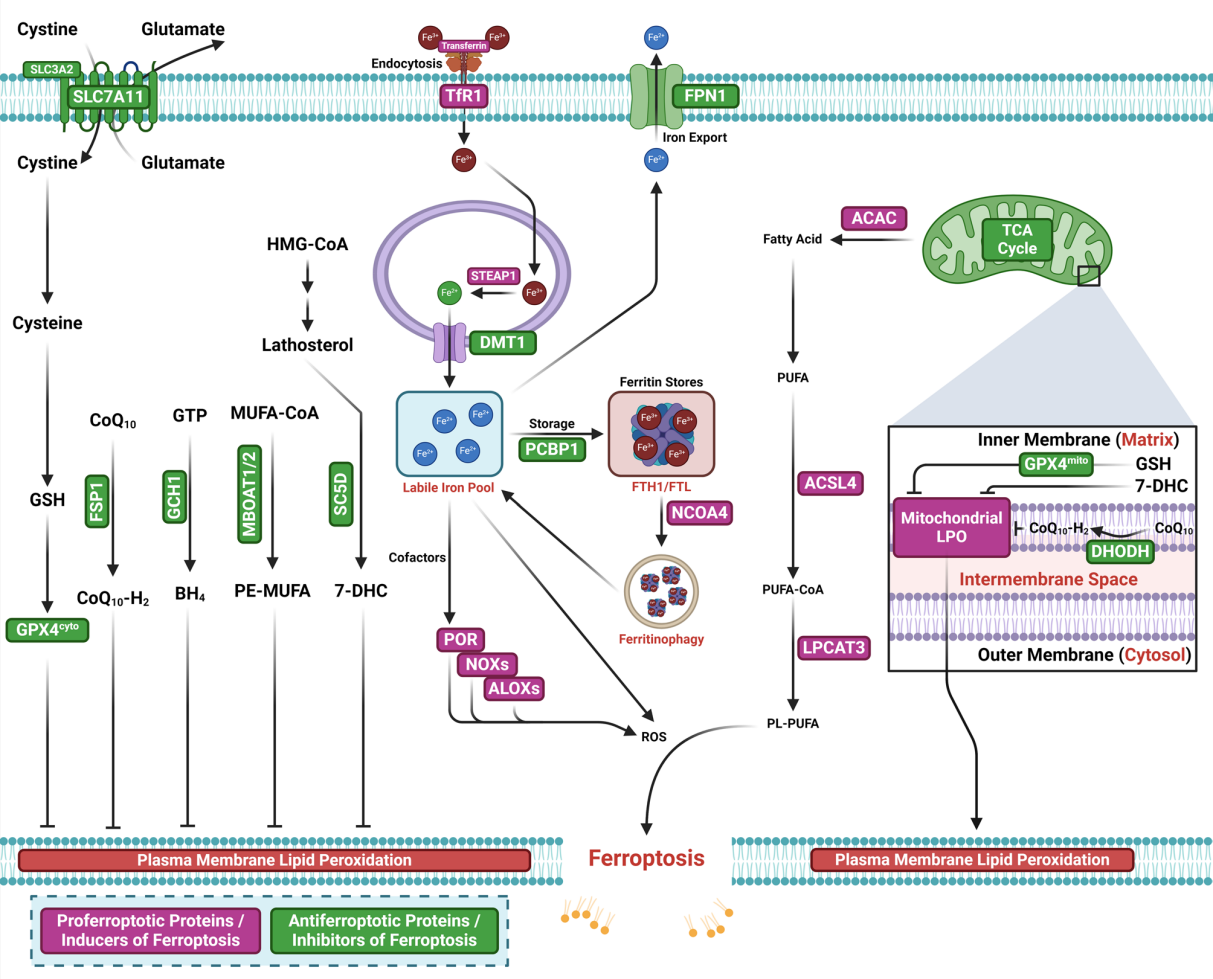
Core mechanisms of ferroptosis. Ferroptosis is induced by an imbalance of pro-ferroptotic factors and anti-ferroptotic defense mechanisms. The SLC7A11 /SLC3A2 system imports cystine for GSH synthesis to quench lipid peroxides, along with other endogenous antioxidant mechanisms: GCH1, FSP1, MBOAT1/2, and SC5D. Iron import and mobilization through POR/NOXs/ALOXs catalyzes ROS synthesis, which may alongside the Fenton reaction induce the synthesis of lipid peroxides. Lipid peroxides accumulate in the plasma membrane and lead to cell lysis

Ferroptosis, a type of non-apoptotic RCD, is characterized by iron-dependent lipid peroxidation (LPO) and a decline in antioxidant capacity [83]. Ferroptosis is a non-apoptotic form of RCD (Fig. 2) [83–85]. Ferroptosis is primarily caused by iron accumulation and LPO, which results in plasma membrane rupture [86]. A subtle imbalance between ferroptosis defense systems and ferroptosis-promoting factors dictates the induction and execution of ferroptosis, which is highly regulated by ferroptosis inducers and inhibitors [31]. The inhibition of the solute carrier family 7 member 11 (SLC7A11)-glutathione (GSH)-glutathione peroxidase 4 (GPX4) system and the accumulation of free iron are the two primary‌ signals that trigger ferroptosis [87]88].

Iron plays a pivotal role in ferroptosis, particularly through its involvement in the production of reactive oxygen species (ROS) and propagation of LPO, which are central to the induction of ferroptosis. Excessive accumulation of free iron within cells contributes to the buildup of lethal lipid peroxides, triggering ferroptosis [87]. Iron facilitates LPO through two distinct pathways. The first is the non-enzymatic Fenton reaction, in which free iron catalyzes the conversion of hydrogen peroxide (H_2_O_2_) into highly reactive hydroxyl radicals (•OH). These radicals initiate LPO by reacting with polyunsaturated fatty acid-containing phospholipids (PUFA-PLs). The second mechanism involves enzymatic processes, where iron functions as a cofactor for specific peroxidases, such as arachidonate lipoxygenases (ALOXs) and cytochrome P450 oxidoreductase (POR), which enhance LPO, ROS production, and PUFA-PL oxidation [31, 88–90]. Iron primarily exists in two oxidation states: ferric (Fe³⁺) and ferrous (Fe²⁺). In its ferrous form, iron reacts with PUFA-PL hydroperoxides (PUFAs-PL-OOH) to produce ^•^OH. These radicals, in turn, attack PUFAs, perpetuating the LPO chain reaction and driving ferroptosis [91]. In the enzymatic LPO pathway, Fe²⁺ acts as an indispensable cofactor for iron-dependent peroxidases, amplifying their activity and initiating PUFA-PL dioxygenation within membranes [92, 93]. This pathway begins with the enzyme ACSL4, which activates free PUFAs by conjugating them with coenzyme A (CoA) to form polyunsaturated fatty acid (PUFA)-CoA molecules. These activated molecules are re-esterified into phospholipids by LPCAT3, which incorporates PUFA-CoAs into membrane phospholipids [94–96]. The resulting PUFA-PLs are subsequently oxidized by iron-dependent enzymes such as ALOXs and PORs, producing PUFA-PL-OOH with the aid of labile iron and molecular oxygen [97–99]. Recent reviews have elaborated the lipid sources and mechanisms underlying ferroptosis in detail [100–102]. In the non-enzymatic LPO pathway, iron drives ferroptosis by initiating direct Fenton reaction-mediated peroxidation of PUFA-PLs [100]. During this process, iron catalyzes the conversion of H₂O₂ into •OH, a highly reactive and mobile form of ROS. These radicals interact with PUFA-PLs to form PUFA-PL-OOH, thereby initiating LPO [103–105]. Once formed, with the help of labile iron, PUFA-PL-OOH can propagate peroxidation to adjacent PUFA-PL molecules, particularly when these peroxides are not efficiently neutralized. Consequently, cellular conditions that increase the labile iron pool (LIP), including suppression of the iron exporter ferroportin [106–108], autophagic degradation of ferritin [109, 110], and heightened transferrin uptake, enhance the susceptibility of cells to ferroptosis [90, 111].

Ferroptosis is regulated by robust antioxidant systems within the cell, and the coupled enzyme-metabolite systems collectively termed ferroptosis defense mechanisms are designed to directly neutralize lipid peroxides and prevent the onset of ferroptosis [56, 112, 113]. Recently, both GPX4-dependent and GPX4-independent pathways with distinct subcellular localizations have been identified as critical regulators of ferroptosis, including the GPX4-GSH axis [31, 114], FSP1-CoQH_2_ system [115–117], GCH1-BH_4_ system [118, 119], DHODH-CoQH_2_ system [120], MBOAT1/2-MUFA system [121], and SC5D-7-DHC axis (Fig. 2) [122, 123].

### Induction of ferroptosis to kill cancer

Ferroptosis has attracted considerable attention in cancer research. Targeting ferroptosis may offer therapeutic strategies for cancer treatment [124, 125]. Ferroptosis acts as an innate tumor suppressor in cancer cells that are usually resistant to apoptosis, and traditional therapies are extremely vulnerable to ferroptosis, underscoring its potential as a treatment approach for cancers, especially refractory cancers [31]. Ferroptosis can be leveraged to kill cancer cells and overcome cancer drug resistance [126–128]. The pharmacological induction of ferroptosis can reverse drug resistance in tumors [128, 129]. Moreover, induction of ferroptosis can reverse resistance to targeted therapies [130, 131] and immunotherapies [132]. In addition, induction of ferroptosis using new methods such as proteolysis-targeting chimeras (PROTACs), sonodynamic therapy (SDT), photodynamic therapy (PDT), and nanomaterials has been attempted for cancer therapy [133, 134]. The combination of ferroptosis and nanotechnology provides effective antitumor responses, and ferroptosis-based nanomedicines offer new strategies for cancer treatment [135, 136]. The combination of nanomedicines and ferroptosis has shown a close relationship with high efficacy in treating various cancer types [134, 136, 137]. Nanomedicines targeting ferroptosis can overcome anticancer resistance [137, 138]. Various ferroptosis nano-inducers have shown an extensive range of functions in reversing resistance to therapy [137, 139].

## Interplay of cGAS-STING and ferroptosis in cancers

### Activation of cGAS-STING triggers ferroptosis in cancers

Emerging evidence has revealed that crosstalk between cGAS-STING and ferroptosis regulates cancer pathogenesis. In 2020, Dang et al. reported that the cGAS-STING1 pathway is involved in mitochondrial DNA (mtDNA) stress-induced ferroptosis in cancer through the activation of autophagy [2]. Nuclear translocation of lysosomal cysteine protease cathepsin B (CTSB) activates the cGAS-STING pathway, which induces autophagy and triggers ferroptosis by degrading GPX4 in human pancreatic cancer cells [2]. Genetic inhibition of CTSB-dependent STING1 activation has been shown to suppress ferroptosis in cell culture and animal models [2]. This observation was corroborated by a study by the same group that reported that cGAS-STING activation contributes to ferroptosis by activating autophagy in cancer [25]. Zalcitabine, an antiviral drug for human immunodeficiency virus infection, has been shown to induce oxidative mtDNA damage and decrease mitochondrial function and cause degradation of the mitochondrial transcription factor TFAM, resulting in the activation of the cGAS-STING pathway, induction of autophagy, and subsequent autophagy-dependent ferroptosis through LPO [25]. A subsequent study replicated these findings [26]. The findings of the subsequent study further demonstrated that STING activation triggers ferroptosis in human pancreatic cancer cell lines by enhancing MFN1/2-dependent mitochondrial fusion and mitophagy-mediated mitochondrial removal [26]. Erastin, a classic ferroptosis inducer, induces the accumulation of STING in mitochondria, where it binds to MFN1/2 to trigger mitochondrial fusion, leading to the subsequent production of ROS and LPO. Loss of STING or MFN1/2 has been shown to decrease the sensitivity of pancreatic cancer cells to ferroptosis in in vitro or xenograft mouse models. These results established a new mitochondrial fusion-dependent mechanism of ferroptosis and highlight a potential strategy for enhancing ferroptosis-based therapy by activating the cGAS-STING pathway [26].

In acute myeloid leukemia (AML), inhibition of phosphoseryl-tRNA kinase (PSTK), a critical enzyme in selenoprotein biosynthesis, has been shown to selectively impair AML growth and eradicate chemoresistant leukemic stem cells while sparing normal hematopoietic cells [27]. Mechanistic studies have revealed that PSTK inhibition triggers mitochondrial ROS accumulation, leading to the release of mtDNA into the cytosol, which activates the cGAS-STING pathway [27]. Activated STING promotes ferritinophagy (via NCOA4), releasing free iron, which exacerbates LPO and ROS generation to ultimately drive ferroptosis [27]. PSTK inhibition synergizes with chemotherapy to eliminate residual chemoresistant cells and reduces relapse in AML models [27]. Genetic or pharmacological inhibition of STING with H151 has been shown to rescue the ferroptotic cell death and ROS accumulation induced by PSTK inhibition, although selenoprotein levels (e.g., GPX4) remain low [27]. Activation of STING with diABZI has been shown to synergize with PSTK inhibition to amplify ROS and ferroptosis [27]. The cGAS-STING pathway amplifies ROS and iron dysregulation, creating a self-reinforcing cycle that culminates in an oxidative crisis and ferroptosis. Thus, although ROS/mtDNA release initiates this cascade, cGAS-STING activation is a critical upstream regulator of ferroptosis. This interplay forms a PSTK-cGAS-STING-ROS loop that is essential for oxidative crisis and ferroptosis in AML [27]. Liao et al. confirmed that USP34 promoted the progression of cervical cancer by upregulating prolyl isomerase 1 (PIN1) expression via SUMOylation, thereby inhibiting ferroptosis by suppressing the cGAS-STING pathway [28]. In summary, activation of the cGAS-STING pathway in tumors can induce ferroptosis, thereby achieving therapeutic effects against cancer. Thus, pharmacological targeting of the cGAS-STING-ferroptosis pathway represents a novel therapeutic strategy for cancer.

This section establishes that activation of the cGAS-STING pathway drives ferroptosis in multiple cancers through interconnected mechanisms: inducing autophagy (degrading GPX4), promoting mitochondrial fusion/mitophagy, and triggering ferritinophagy. Despite the establishment of cGAS-STING as a critical ferroptosis regulator, key questions remain unresolved, including the context-dependent molecular switches that determine whether pathway activation promotes ferroptosis versus other outcomes (e.g., inflammation and senescence) across cancer types and the influence of chronic versus acute STING activation on therapeutic efficacy and resistance. Future studies should aim to delineate tissue-specific regulatory mechanisms, identify biomarkers predicting ferroptotic responses to STING modulation, and develop optimized combinatorial strategies (e.g., with chemotherapy, immunotherapy, or other ferroptosis inducers) that can maximize tumor-selective ferroptosis while minimizing systemic toxicity in preclinical and clinical settings.

### Induction of ferroptosis activates cGAS-STING in cancers

Ferroptosis has been identified as an upstream event that activates the cGAS/STING pathway in cancer [29]. This study also demonstrated that ferroptosis and radiotherapy (RT)-induced formation of dsDNA are parallel upstream events that converge upon cGAS-STING activation [29]. The findings of this study further demonstrated that BIBR1532, a telomerase inhibitor, synergizes with radiotherapy (RT) to enhance radiosensitivity and antitumor immunity in non-small cell lung cancer (NSCLC) through two interconnected mechanisms: inhibition of DNA damage repair and induction of ferroptosis [29]. RT induces DNA double-strand breaks (DSBs), while BIBR1532 suppresses ATM /ATR/CHK1-mediated DNA repair pathways, leading to persistent cytoplasmic accumulation of dsDNA, which activates the cGAS-STING pathway and triggers type I IFN-β production, dendritic cell (DC) maturation, and CD8⁺ T cell -mediated adaptive immunity [29]. BIBR1532 amplifies RT-induced ferroptosis, as evidenced by increased LPO, ROS accumulation, and Fe²⁺ overload while downregulating anti-ferroptosis proteins (e.g., GPX4, SLC7A11). Ferroptosis causes mitochondrial stress, releasing mtDNA into the cytoplasm, which further activates cGAS-STING to create a positive feedback loop for immune activation [29]. However, this study did not support reverse causality (i.e., cGAS -STING directly induces ferroptosis). Instead, it suggested that ferroptosis-associated mitochondrial dysfunction and ROS production create conditions that enhance cGAS -STING signaling [29].

Transforming growth factor-beta-activated kinase 1 (TAK1) deficiency in hepatocytes induces ferroptosis through oxidative stress and iron dysregulation [30]. Ferroptosis causes oxidative DNA damage (e.g., 8-OHdG), and the damaged DNA is released into the extracellular space. The damaged DNA activates macrophage cGAS -STING signaling, driving inflammation, fibrosis, and liver tumorigenesis. Inhibiting ferroptosis (with Fer-1) or blocking STING (with C-176) has been shown to attenuate liver injury and tumor progression [30]. Blocking ferroptosis (through Fer-1) has been shown to suppress cGAS-STING activation in macrophages. STING inhibition does not reverse ferroptosis, but mitigates its downstream inflammatory effects, indicating that ferroptosis precedes STING activation [30]. Targeting ferroptosis or cGAS-STING can synergistically treat diseases involving oxidative stress, chronic inflammation, and cancer [30]. Taken together, these results indicate that ferroptosis in tumors can activate the cGAS-STING pathway, thereby exerting therapeutic effects against cancer cells. Thus, the pharmacological targeting of the ferroptosis-cGAS -STING pathway may serve as a novel therapeutic strategy for tumor treatment. On the basis of these findings, drug-targeted modulation of the crosstalk between cGAS -STING and ferroptosis has emerged as a novel approach for cancer therapy.

Emerging evidence has shown that ferroptosis is an upstream activator of the cGAS-STING pathway in multiple cancers. Ferroptosis-induced mitochondrial or oxidative DNA damage directly triggers cytoplasmic cGAS-STING activation, which drives antitumor immunity or inflammation/fibrosis. Critically, ferroptosis initiates a self-reinforcing cycle, in which subsequent cGAS-STING signaling amplifies immune or inflammatory responses. Combined treatment with ferroptosis inducers and STING agonists has demonstrated synergistic therapeutic potential.

Key unresolved questions include defining the precise molecular sensors and spatiotemporal dynamics linking distinct ferroptosis drivers (e.g., LPO and iron overload) to specific damage-associated molecular patterns (DAMPs; mtDNA vs. nuclear DNA) that activate cGAS-STING and elucidating why this axis drives antitumor immunity in some contexts but pathogenic inflammation in others. Future studies must also aim to identify biomarkers predicting directional fidelity (ferroptosis→STING vs. STING→ferroptosis) across tissues, delineate mechanisms of crosstalk with other cell death or DNA repair pathways, and develop optimized combinatorial regimens (e.g., ferroptosis inducers + immunotherapy/STING modulators) that can maximize tumor-specific immune activation while avoiding pro-tumorigenic inflammation in translational models.

## Nanomedicines can target the interplay of cGAS-STING and ferroptosis in cancers

### Nanomedicines can activate cGAS-STING to induce ferroptosis

**Fig. 3:**
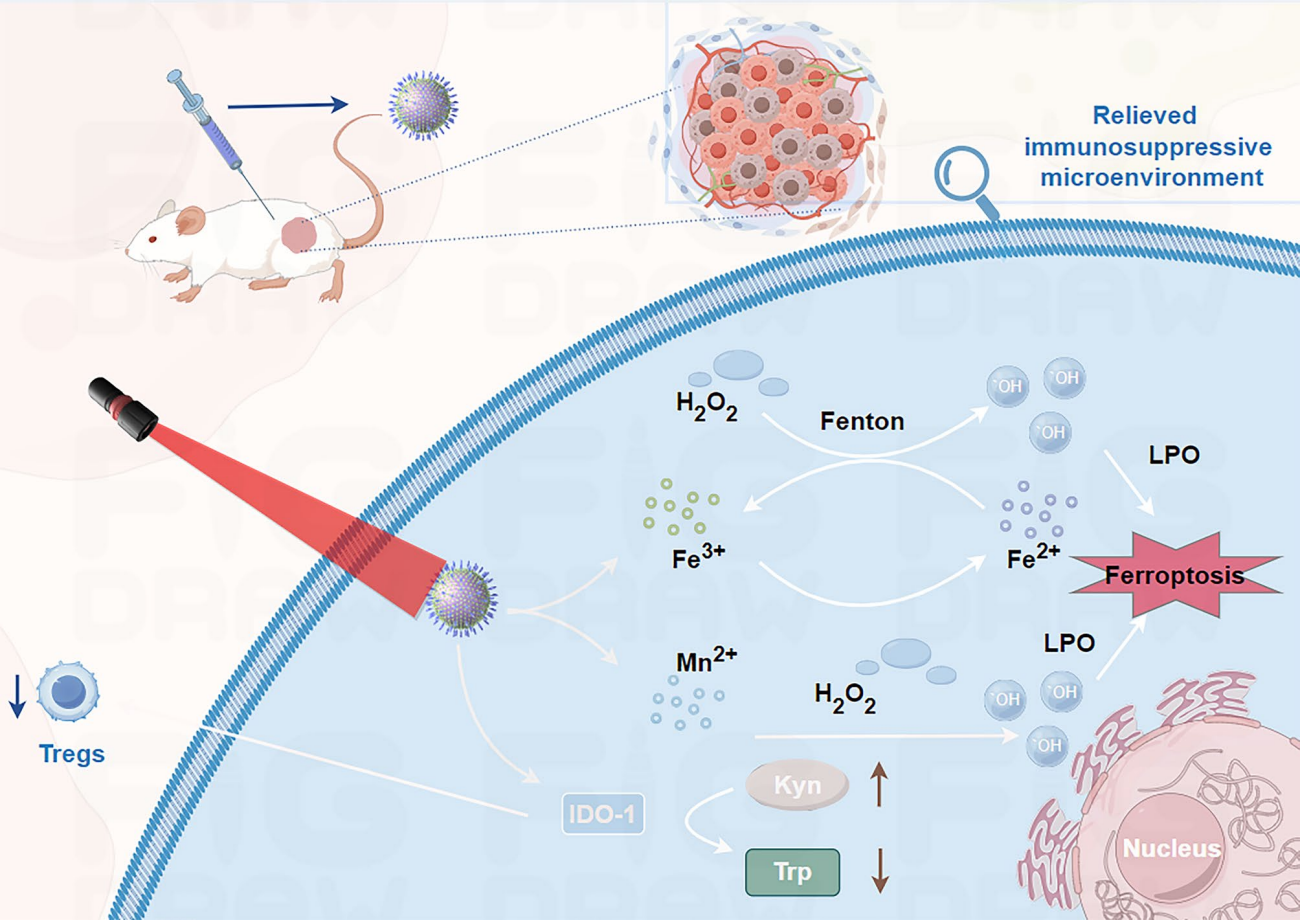
Schematic illustration of the synthesis and therapeutic mechanism of manganese-based nanoenzyme TME-responsive Mn(III)-SS NEs for ferroptosis -enhanced ICD and immune-driven ferroptosis

A manganese-based nanoenzyme (Mn(III)-SS NEs) was developed to synergize ferroptosis induction and immunotherapy for enhanced breast cancer theranostics [36] (Fig. 3; Table 1). This nanoplatform leverages triple tumor microenvironment (TME)-responsive mechanisms by GSH depletion, ROS generation, cGAS-STING activation, and an immune-driven ferroptosis loop [36]. Mn(III)-SS NEs consume intracellular GSH through disulfide-thiol exchange and Mn³⁺/Mn²⁺ redox reactions, inhibiting GPX4 activity [36]. Concurrently, Mn²⁺ catalyzes a Fenton-like reaction with H₂O₂ to produce ^•^OH, exacerbating LPO and ferroptosis [36]. Mn²⁺ released from the nanoplatform activates the cGAS-STING pathway, promoting dendritic cell maturation and CD8⁺ T cell infiltration. This enhances IFN-γ secretion, which downregulates SLC7A11 (a cystine/glutamate transporter), further reducing GSH synthesis and amplifying ferroptosis [36]. Ferroptosis-induced immunogenic cell death (ICD) releases DAMPs (e.g., HMGB1 and CRT), recruiting more CD8⁺ T cells to sustain IFN-γ production. This creates an antitumor immune “closed loop,” effectively inhibiting both primary and distant tumors [36]. The nanoplatform significantly suppressed tumor growth and prolonged survival in 4T1 tumor-bearing mice. Mn(III)-SS NEs represent a multifunctional theranostic platform for cGAS -STING activation-mediated induction of ferroptosis to immune activation. This strategy highlights the potential of metal-based nanoenzymes for precise, immune -enhanced cancer treatment [36].

**Table 1:** Overview of nanoparticles activate cGAS-STING to induce ferroptosis in cancer treatment

Nanoparticles	Nanomaterials composition	Cancer	Tested model	Effects OR Involved mechanism	Ref
Mn(III)-SS NEs		Breast cancer	4T1 tumor-bearing mice	ZCUNH synergize ferroptosis induction to boost immunotherapy by activating cGAS-STING pathway in osteosarcoma	[36]

U-104,a novel carbonic anhydrase inhibitor

### Nanomedicine induce ferroptosis to activate cGAS-STING

In this section, we describe how rationally designed nanomaterials, from metal-organic frameworks to exosome hybrids, orchestrate ferroptosis induction and subsequent STING activation through spatially controlled ion release, redox disruption, and DAMP generation (Table 2). These systems not only amplify ICD, but also reprogram the tumor microenvironment, transforming transient cytotoxic responses into sustained antitumor immunity. The following synthesis highlights the material innovations, mechanistic convergence, and therapeutic synergy, thereby defining this rapidly evolving frontier.

**Table 2:** Overview of nanoparticles induce ferroptosis to activate cGAS-STING in cancer treatment

Nanoparticles	Loaded agent	Cancer	Tested model	Effects OR Involved mechanism	Ref
Cu/ZIF-8@U-104@siNFS1-HA		Osteosarcoma	MG-63 tumor-bearing C57BL/6J mice	ZCUNH synergize ferroptosis induction to boost immunotherapy by activating cGAS-STING pathway in osteosarcoma.	[37]
DHA@MIL-101	U-104	NSCLC	Lewis lung cancer cells tumor-bearing C57 mice	DHA@MIL-101 enhances anti-cancer immunotherapy by inducing Ferroptosis to activate cGAS-STING for reprogramming of macrophage.	[38]
Exo@MnIO&BG	Dihydroartemisinin	Breast cancer	4T1 tumor-bearing mice	Exo@MnIO&BG enhances anti-cancer immunotherapy by boosting ferroptosis to activate cGAS-STING.	[140]
Fe-THBQ/SR	SR-717	Breast cancer	4T1 tumor-bearing mouse	Fe-THBQ/SR promotes tumor immunotherapy via synergistic ferroptosis and STING activation.	[141]
CMS	Ca²⁺ & Mn	TNBC	4T1-tumor-bearing mice	The CMS-induced ferroptosis- and Mn²⁺-mediated STING pathway activation promoted tumor -associated macrophage (TAM) polarization toward antitumor phenotypes and dendritic cell (DC) maturation for antigen presentation.	[142]
MG-LAAO	Gallium -magnesium	Breast cancer	4T1-tumor-bearing mice	MG-LAAO blocks tumor cell autophagy, neutralizes acidic TME via H⁺ scavenging, and inhibits lactic acid accumulation, thereby dismantling cytoprotective mechanisms and preventing immune evasion.	[143]
MMP NDs	Mo/Mn	CRC	CT26 tumor bearing mice.	MMP NDs exploit the synergistic redox activity of high-valence Mo/Mn to deplete tumor-overexpressed glutathione (GSH), inducing ferroptosis while activating the cGAS-STING pathway to amplify antitumor immunity. This dual mechanism triggers a self-reinforcing therapeutic cycle: ferroptosis releases tumor antigens, activating CD8⁺ T cells to secrete interferon-γ (IFN-γ), which suppresses GPX4 expression and exacerbates lipid peroxidation, thereby sustaining ferroptotic cell death.	[144]
BQR@MLipo	Brequinar	Breast cancer	4T1-tumor-bearing mice	BQR@MLipo inhibit DHODH to trigger mitochondrial LPO and ROS overproduction, resulting in ferroptosis. This process releases immunogenic damage-associated molecular patterns (DAMPs), including calreticulin (CRT), ATP, and HMGB1, while mitochondrial DNA (mtDNA) leakage activates the cGAS-STING pathway to stimulate IFN-β secretion.	[145]
HBMn-FA	Hemin, BSO and Mn2+	Breast cancer	4T1-tumor-bearing mice	HBMn-FA nanoplatform drives ferroptosis in tumor cells, generating high levels of ROS that induce mitochondrial stress and release endogenous mtDNA, which synergizes with Mn²⁺ to activate the cGAS-STING pathway, while tumor-derived cytosolic dsDNA from ferroptotic cell debris further amplifies STING signaling in dendritic cells (DCs) and other antigen-presenting cells.	[146]
HMG nanoparticles		Breast cancer	4T1-tumor-bearing mice	HMG induces ferroptosis to intensify DNA damage in 4T1 breast cancer cells and synergizes with radiotherapy to activate the Mn²⁺-mediated cGAS-STING pathway, triggering robust systemic antitumor immunity.	[147]

DHODH, dihydroorotate dehydrogenase; U-104,a novel carbonic anhydrase inhibitor

**Fig. 4:**
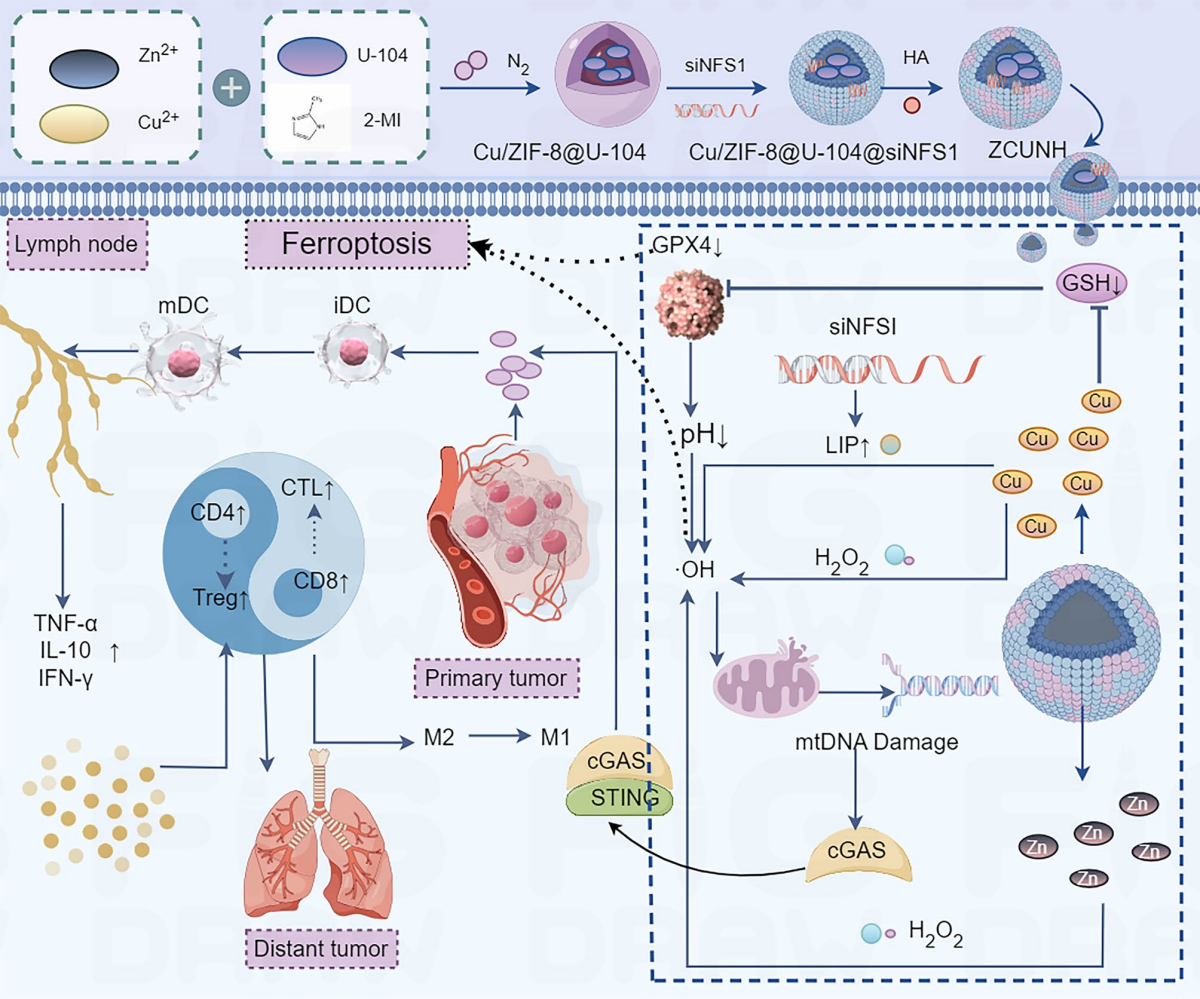
Schematic illustration of the synthetic process and therapeutic mechanism of ZCUNH

A bimetallic nanoplatform (ZCUNH) was synthesized in three steps, i.e. Cu²⁺-doped ZIF-8 nanoparticles (NPs) were synthesized with a one-pot method using a metal-organic framework (MOF) template, with U-104 (a CA IX inhibitor) loaded into the porous structure. siRNA targeting NFS1 (cysteine desulfurase) was then adsorbed onto the NPs through electrostatic interactions. Hyaluronic acid (HA) was coated onto the surface to stabilize siNFS1 through hydrogen bonding and van der Waals forces, thereby enhancing tumor targeting through CD44 receptor binding (Fig. 4; Table 2) [37]. Co-suppression of carbonic anhydrase (CA) IX and cysteine desulfurase (NFS1) increases the LIP and acidifies the TME, enhancing Fe²⁺-driven Fenton reactions. Cu²⁺ depletes GSH, reduces GPX4 activity, and generates •OH, amplifying LPO and ferroptosis [37]. Zn²⁺ released from ZCUNH upregulates cGAS -STING expression, which cooperates with ferroptosis to trigger ICD and remodel the immunosuppressive TME. Ferroptosis-induced DNA damage and DAMPs release promote DC maturation, M1 macrophage polarization, and cytotoxic T cell infiltration, leading to robust antitumor immunity. This study established ferroptosis as an upstream event that activated the cGAS-STING pathway [37]. ZCUNH synergizes with ferroptosis induction to boost immunotherapy by activating the cGAS-STING pathway in osteosarcoma [37]. This study suggests that cGAS-STING activation acts downstream to amplify ICD and antitumor immunity. Thus, targeting ferroptosis -cGAS-STING crosstalk offers a dual-pronged strategy for enhancing both tumor cell death and antitumor immunity, addressing the limitations of conventional therapies.

**Fig. 5:**
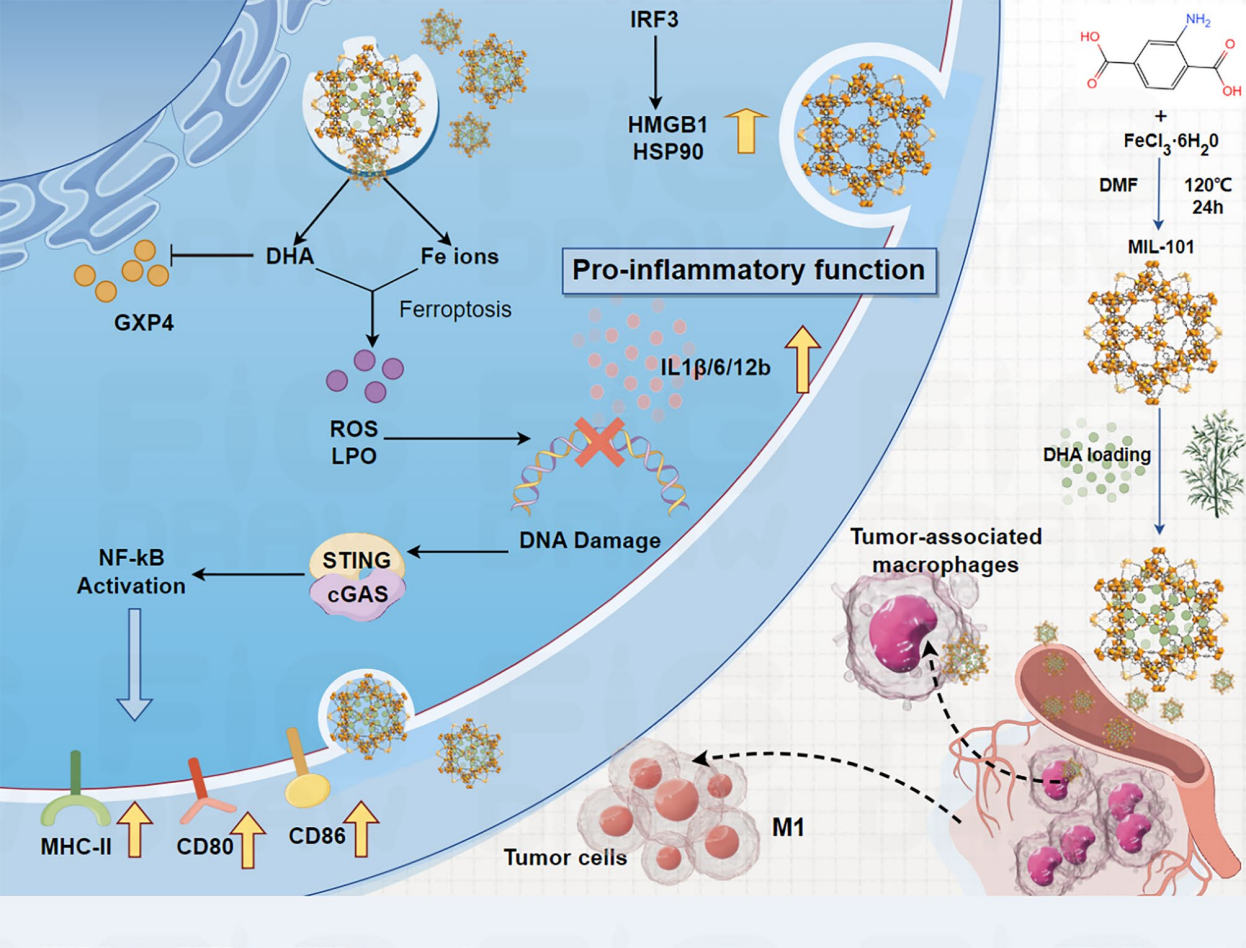
Schematic diagram of DHA nanoreactor-triggered M1 polarization of TAMs via ferroptosis-driven activation of the cGAS/STING signaling pathway. (1) Low-dose DHA iron-based nanoreactors enrich in lung cancer and are subsequently taken up by TAMs. (2) Collapse of the DHA nanoreactor releases iron ions and DHA (which binds and inhibits GPX4), synergistically inducing ferroptosis. (3) Ferroptosis products damage DNA, activating the STING signaling pathway to drive TAM polarization into the M1 phenotype. (4) M1-mediated anti-lung cancer immune effects are thereby initiated

An iron-based MOF nanoreactor, DHA@MIL-101, was successfully developed for encapsulation of dihydroartemisinin (DHA). DHA@MIL-101 exhibited high uptake by tumor-associated macrophages (TAMs) in the TME while preserving their viability (Fig. 5) [38]. DHA@MIL-101 selectively accumulated in tumor tissues and was preferentially phagocytosed by TAMs over cancer cells, leveraging the enhanced permeability and retention (EPR) effect and macrophage phagocytic activity [38]. DHA@MIL-101 triggered ferroptosis in TAMs through iron ion release and GPX4 inhibition. Degradation of the iron-based MOF in the acidic TAM microenvironment released iron ions [38]. DHA directly binds to and inhibits GPX4. Ferroptosis -induced DNA damage in TAMs activated the cGAS-STING pathway, leading to NF-κB and IRF3 activation. DHA@MIL-101 effectively reprogrammed TAMs into antitumor M1 macrophages through ferroptosis-driven activation of the cGAS-STING pathway [38]. This dual mechanism combines direct tumor cell killing (through iron overload in cancer cells) and immune modulation (through TAM reprogramming) [38]. In contrast, blocking ferroptosis impaired DHA@MIL-101-induced activation of STING signaling and phenotypic remodeling. This study highlights the potential of nanocarrier-phytopharmaceutical hybrids to modulate the tumor immune microenvironment, offering a novel strategy for cancer immunotherapy. These results provide a paradigm for leveraging ferroptosis and innate immune cGAS-STING pathways to enhance antitumor immunity [38].

**Fig. 6:**
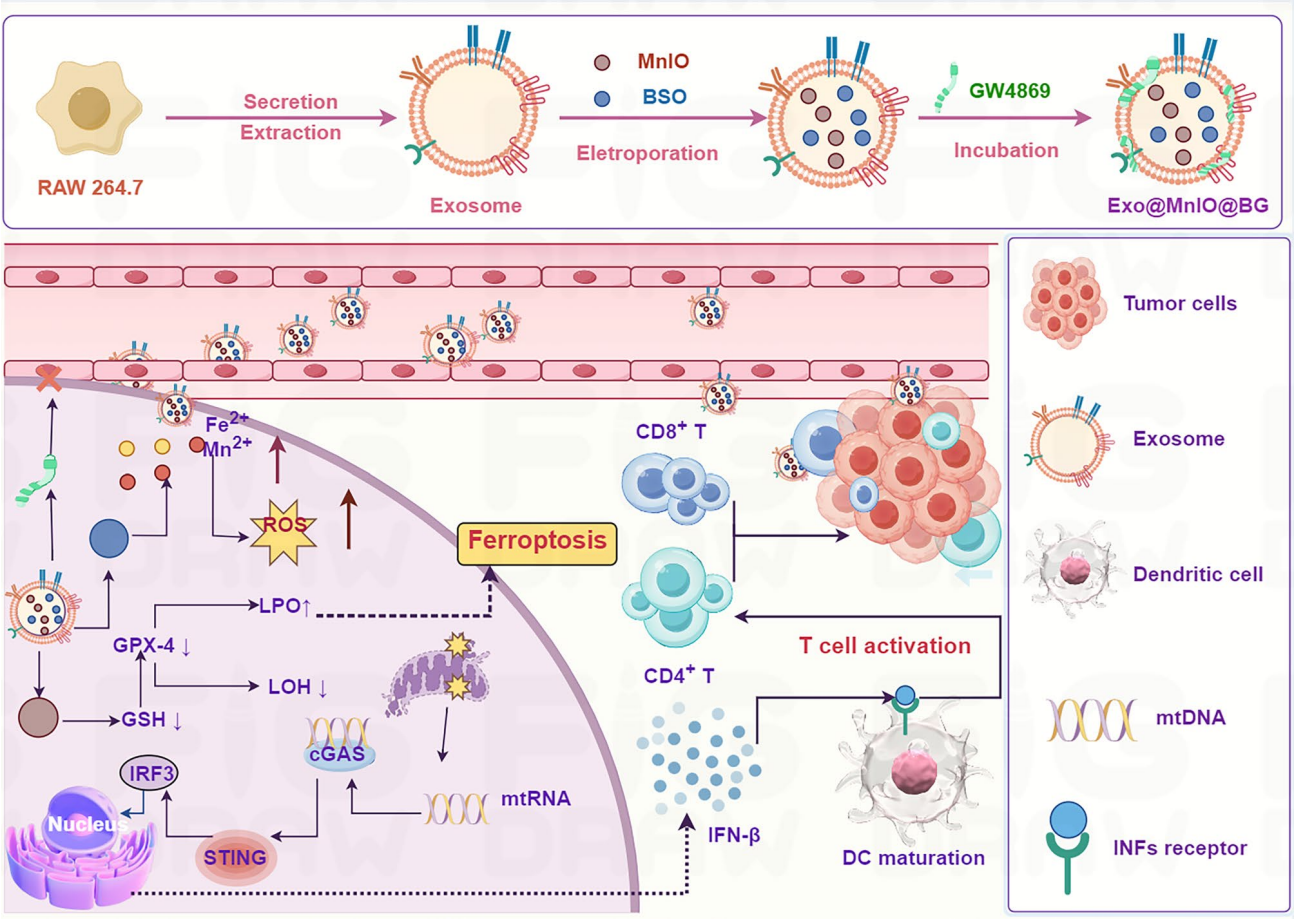
Schematic illustration of the preparation process of Exo@MnIO&BG. Therapeutic mechanism of Exo@MnIO&BG for ferroptosis-based immunotherapy

A multifunctional exosome-based platform co-loaded with manganese-doped iron oxide nanoparticles (MnIOs), the exocytosis inhibitor GW4869, and the glutathione synthesis blocker l-buthionine sulfoximine (BSO) was engineered to synergistically disrupt iron metabolism and redox homeostasis and thereby amplify tumor immunotherapy (Fig. 6) [140]. By leveraging the exosomes’ efficient delivery of MnIO and GW4869-mediated suppression of iron efflux, the system achieved remarkable iron retention in tumors [140]. This iron overload, combined with the BSO-induced glutathione depletion and elevated oxidative stress, drove robust ferroptosis in cancer cells. The dual homeostasis-disruption strategy not only amplified LPO but also activated the cGAS-STING pathway, triggering potent antitumor immunity [140]. In orthotopic breast cancer models, this engineered exosome platform demonstrated superior efficacy in suppressing primary tumor progression and pulmonary metastasis, establishing a paradigm for the precise modulation of tumor microenvironmental vulnerabilities to enhance immunotherapeutic outcomes [140].

**Fig. 7:**
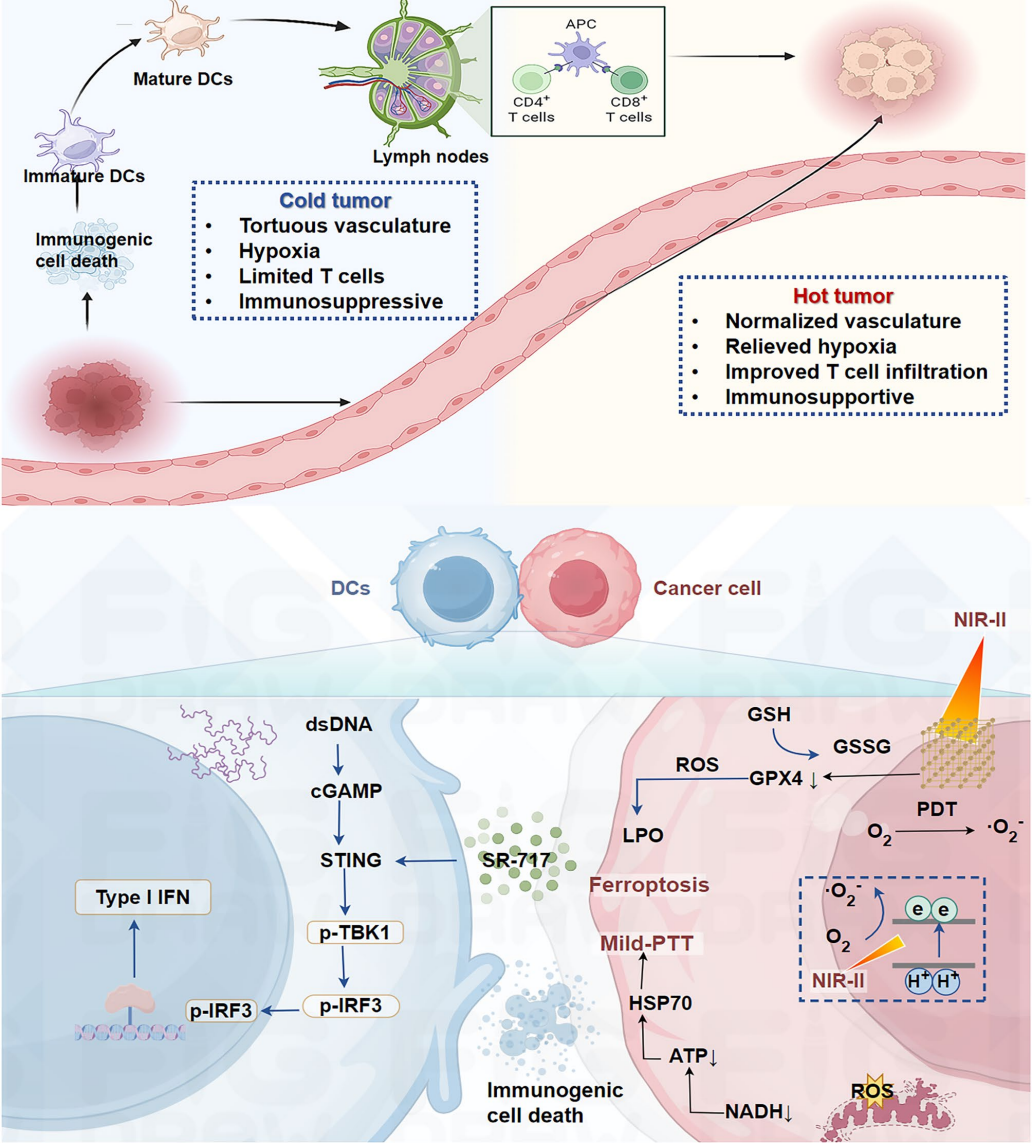
Schematic illustration of Fe-THBQ/SR as an NIR-II PDT and NIR-II mild-PTT agent under 1064 nm laser irradiation, inducing ferroptosis, STING activation, and vasculature normalization

A multifunctional nanoplatform (Fe-THBQ/SR) was engineered by encapsulating the STING agonist SR-717 into an iron-tetrahydroxy-1,4-benzoquinone (Fe-THBQ) MOF (Fig. 7) [141]. Under 1064-nm laser irradiation, Fe-THBQ demonstrated exceptional efficacy as an NIR-II photosensitizer, generating substantial ROS, which downregulated heat shock protein (HSP) expression to enable mild photothermal therapy (mild-PTT) and triggered ferroptosis through GSH depletion and GPX4 inactivation [141]. Meanwhile, Fe-THBQ/SR exhibited GSH-responsive release of SR-717, which synergistically amplified STING activation by coupling with ROS-induced double-stranded DNA damage [141]. This dual-action mechanism promoted tumor vasculature normalization and alleviated hypoxia, significantly enhancing the efficacy of PDT [141]. Remarkably, the single-laser-triggered nanoplatform achieved NIR-II PDT, NIR-II mild-PTT, and STING pathway activation in a reinforcing cycle. Together, these synergistic effects elevated tumor immunogenicity, reprogrammed the immunosuppressive TME, and boosted cytotoxic T lymphocyte infiltration, ultimately improving therapeutic outcomes [141]. These findings highlight the use of Fe-THBQ/SR as a pioneering strategy for integrating multimodal tumor ablation with immune modulation, offering a robust solution for overcoming resistance in cancer therapy [141].

**Fig. 8:**
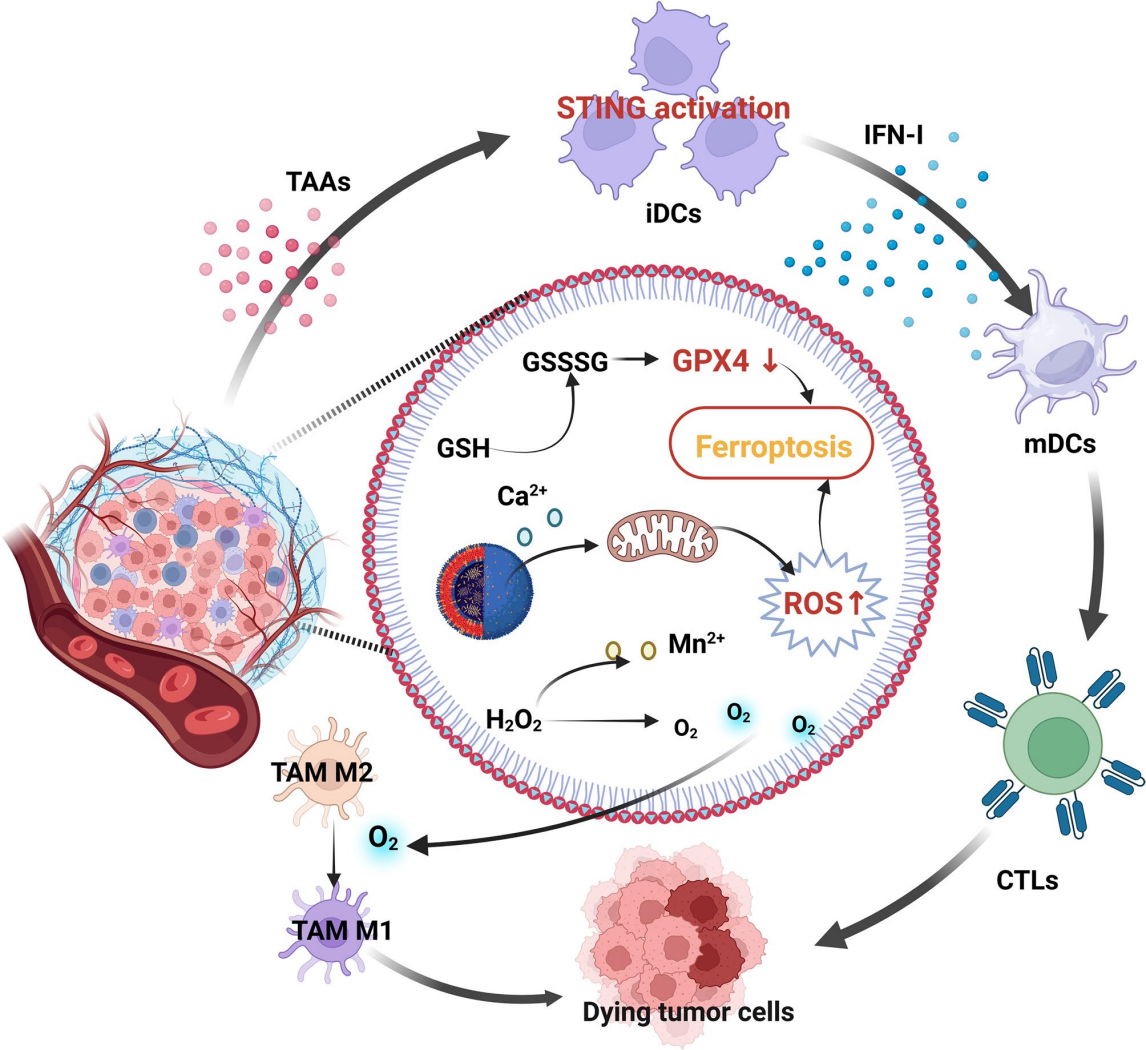
Schematic illustration of the nanostimulator (CMS) inducing tumor cell ferroptosis while simultaneously awakening TAMs and DCs to boost anti-tumor immune responses

A Ca²⁺- & Mn-based dual-ion hybrid nanostimulator (CMS) has been shown to function as a dual-functional agent for triple-negative breast cancer (TNBC) therapy by enhancing antitumor immunity through induction of ferroptosis to activate innate immune responses (Fig. 8) [142]. The mixed-valence manganese in CMS drives GSH depletion and ROS generation, while its role as an exogenous Ca²⁺ source triggers mitochondrial Ca²⁺ overload, and the consequent amplification of oxidative stress collectively inactivates GPX4 and induces lethal LPO, culminating in robust ferroptosis in cancer cell [142]. The induced ferroptosis- and Mn²⁺-mediated STING pathway activation promoted TAM polarization toward antitumor phenotypes and DC maturation for antigen presentation [142]. This dual-action strategy fosters tumor -specific cytotoxic T lymphocyte (CTL) infiltration and establishes a potent adaptive immune response [142]. Collectively, CMS represents a groundbreaking approach that integrates ferroptosis induction with innate immune activation, thereby offering a transformative paradigm for TNBC immunotherapy [142].

**Fig. 9:**
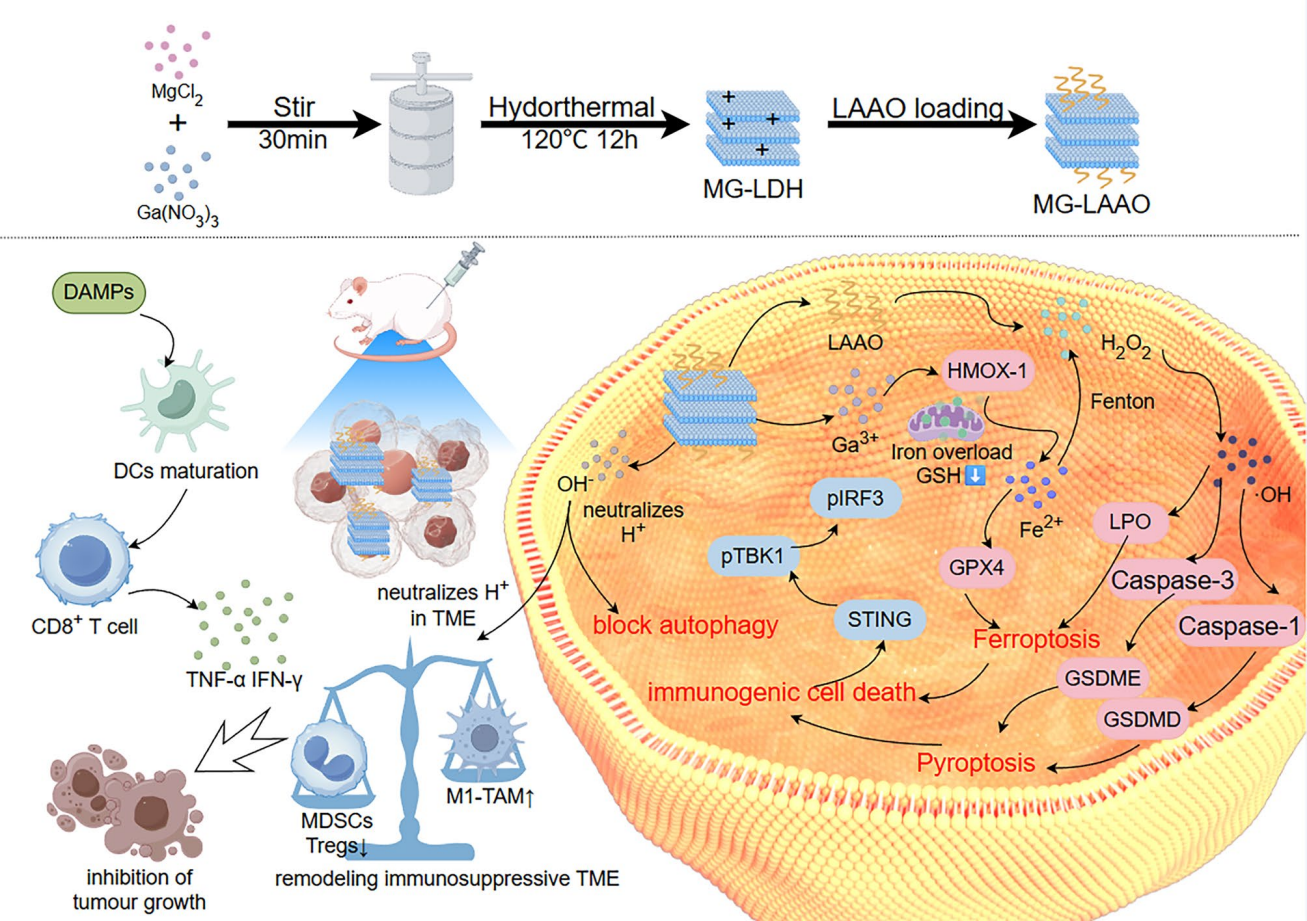
Schematic diagram of MG-LAAO action: Degradation in the mildly acidic TME neutralizes H⁺ and inhibits tumor cell autophagy, concurrently releasing Ga³⁺ and LAAO. Ga³⁺ upregulates HMOX-1, causing mitochondrial iron overload and Fe³⁺ release. Fe³⁺ reacts with GSH, reducing to Fe²⁺ which downregulates GPX4 and participates in the Fenton reaction. This consumes LAAO-produced H₂O₂, generating cytotoxic ROS and promoting LPO accumulation, ultimately triggering ferroptosis. Additionally, MG-LAAO induces pyroptosis via canonical (caspase-1/GSDMD) and non-canonical (caspase-3/GSDME) pathways, further promoting ICD, activating the STING pathway, and enhancing anti-tumor immunity

Current tumor immunotherapies face several challenges, including poor immunogenicity, an immunosuppressive TME, and cytoprotective mechanisms that dampen immune activation. l-amino acid oxidase (LAAO)-loaded gallium -magnesium layered double hydroxide (MG-LAAO) was engineered to address these limitations by amplifying antitumor immunity through multi-pathway synergy (Fig. 9) [143]. MG-LAAO induces pyroptosis through the caspase-1/GSDMD and caspase-3/GSDME pathways, triggering ICD, while the released Ga³⁺ disrupts mitochondrial iron homeostasis to drive ferroptosis [143]. Concurrently, MG-LAAO blocks tumor cell autophagy, neutralizes acidic TME by H⁺ scavenging, and inhibits lactic acid accumulation, thereby dismantling cytoprotective mechanisms and preventing immune evasion [143]. These coordinated actions robustly activate the cGAS-STING pathway, generating potent systemic antitumor immunity. These results underscore the critical interplay of autophagy inhibition, pyroptosis, ferroptosis, and ICD in reshaping the immunosuppressive TME and elevating immunogenicity [143].

**Fig. 10:**
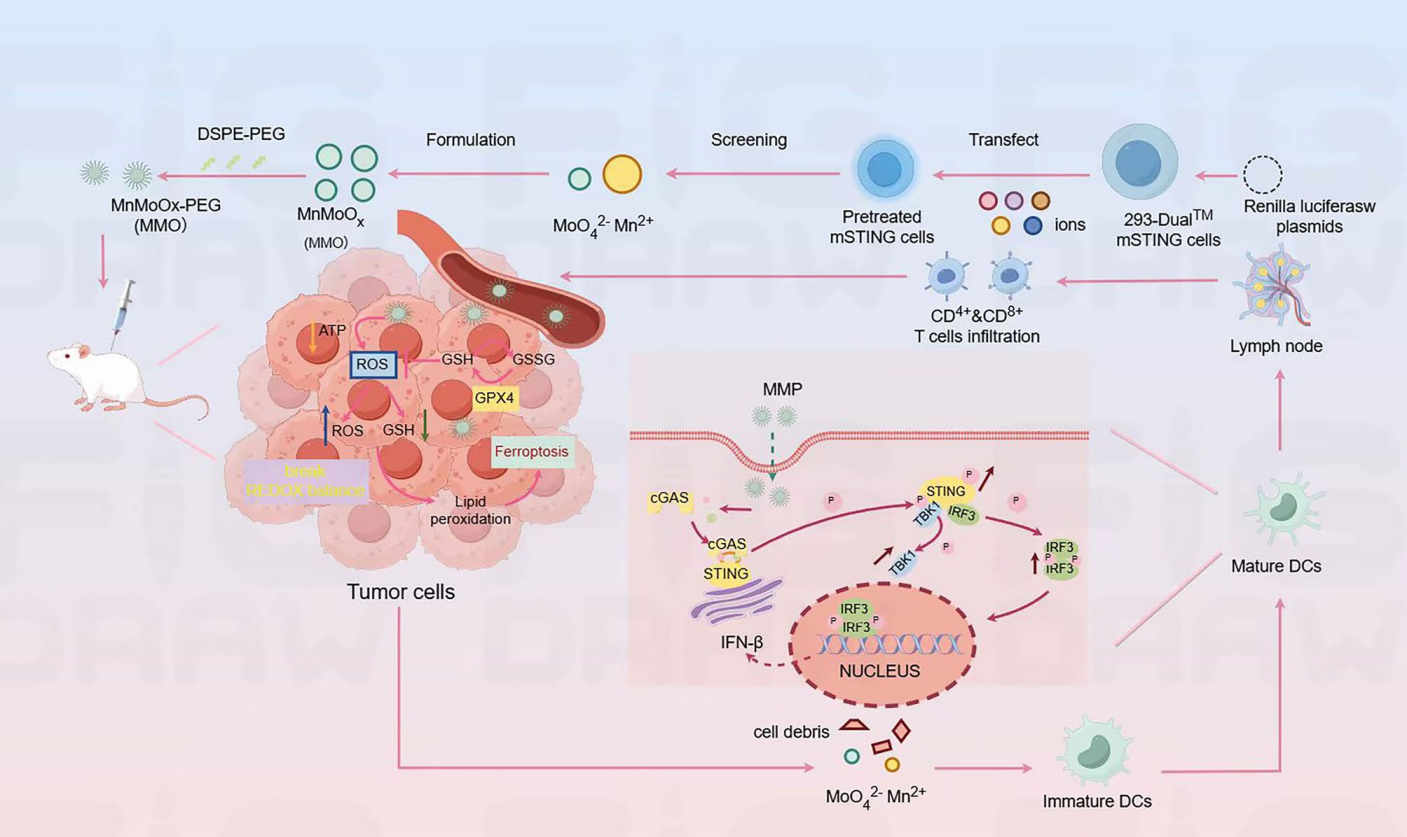
MMP NDs with dual cGAS-STING activation for cancer metalloimmunotherapy. Metal cations and anions exhibiting the strongest cGAS -STING activation were screened using 293-Dual™ mSTING cells and utilized to construct bimetallic oxide MMO NDs; MMP NDs were then obtained by DSPE-PEG₅ₖ modification. MMP NDs containing Mn²⁺ and MoO₄²⁻ effectively activate cGAS-STING. Following systemic administration, MMP NDs consume highly expressed tumor GSH, disrupt redox balance, inhibit GPX4 activity, and subsequently induce tumor cell ferroptosis. Tumor debris containing MMP NDs then triggers tumor-specific cellular immunity

Lei et al. developed polyethylene glycol-modified manganese molybdate nanoparticles (MMP NDs) as a novel metalloimmunotherapy platform for cancer treatment (Fig. 10) [144]. MMP NDs exploit the synergistic redox activity of high-valence Mo/Mn to deplete tumor-overexpressing GSH, induce ferroptosis, and activate the cGAS-STING pathway to amplify antitumor immunity [144]. This dual mechanism triggers a self-reinforcing therapeutic cycle: ferroptosis releases tumor antigens, activating CD8⁺ T cells to secrete interferon-γ (IFN-γ), which suppresses GPX4 expression and exacerbates LPO, thereby sustaining ferroptotic cell death [144].

**Fig. 11:**
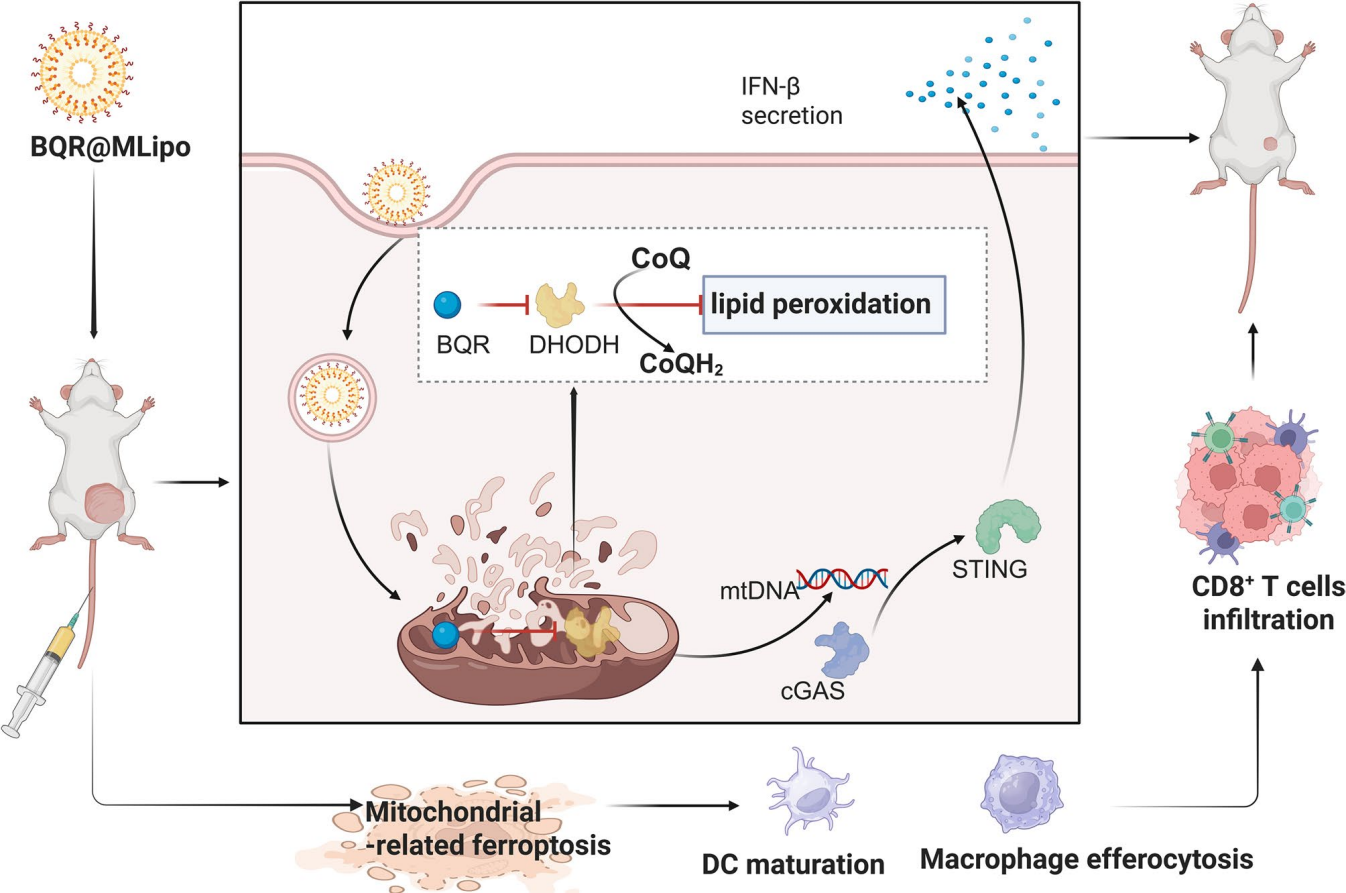
Mechanism of BQR@MLipo for enhancing mitochondria-related ferroptosis to potentiate checkpoint blockade immunotherapy

A mitochondrial-targeted liposomal system loaded with brequinar (BQR@MLipo) was engineered to amplify mitochondrial ferroptosis for in situ bladder cancer therapy (Fig. 11) [145]. BQR@MLipo selectively accumulates in mitochondria, to inhibit dihydroorotate dehydrogenase (DHODH). This process releases immunogenic DAMPs, including calreticulin (CRT), ATP, and HMGB1, while mitochondrial DNA (mtDNA) leakage activates the cGAS -STING pathway to stimulate IFN-β secretion [145]. This immunomodulation enhances dendritic cell-mediated antigen cross-presentation and macrophage phagocytosis [145]. In vivo, BQRs@MLipo demonstrated preferential tumor accumulation, driving robust CD8 + T cell infiltration into the bladder TME, and achieving potent growth suppression [145]. These results provide evidence for the first time that DHODH inhibition induces mitochondrial ferroptosis in bladder cancer immunotherapy, establishing BQR@MLipo as a clinically translatable strategy to synergize ferroptotic tumor elimination with antitumor immunity activation.

**Fig. 12:**
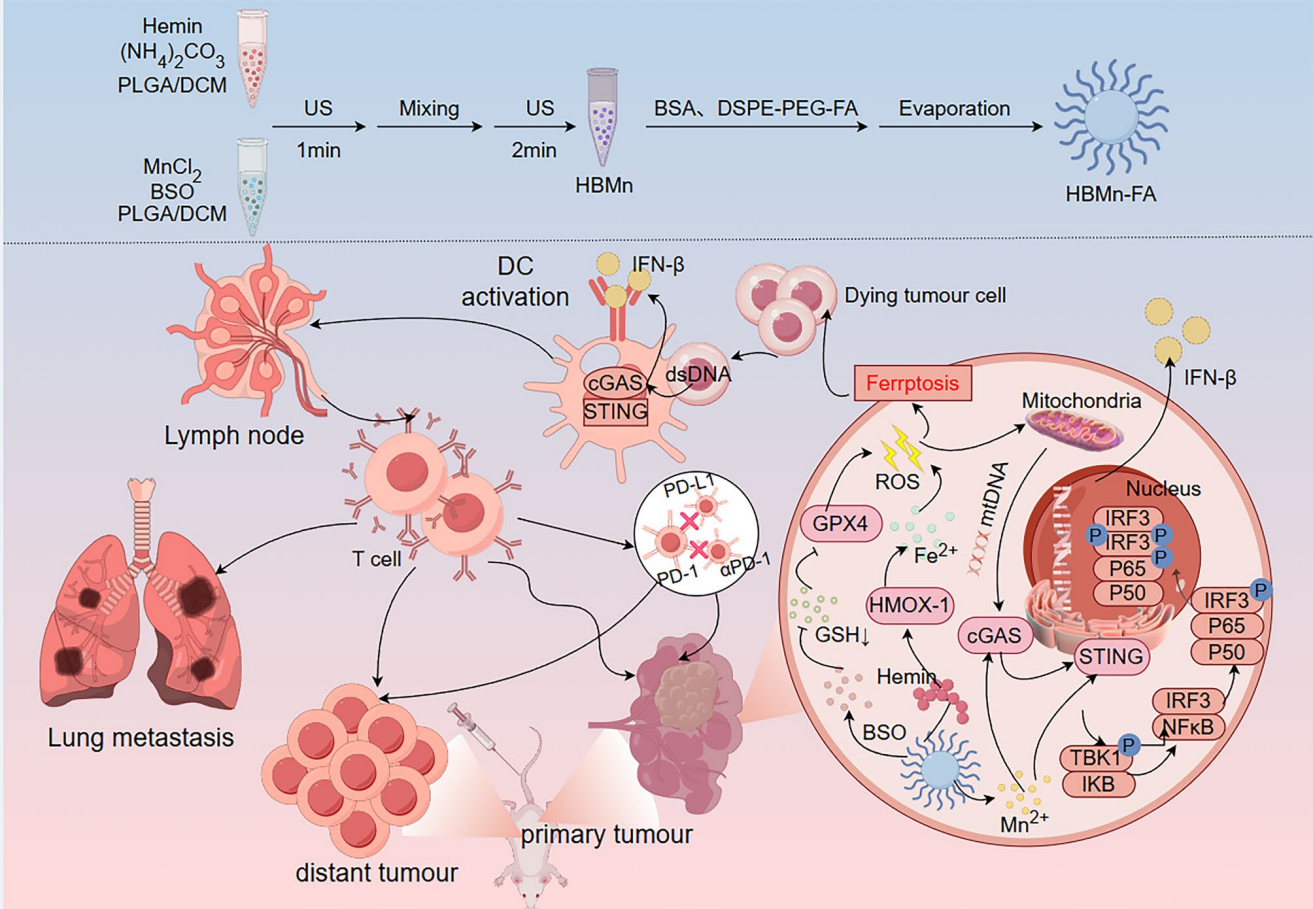
Schematic of the synthesis and cGAS-STING pathway activation mechanism of HBMn-FA. **(a)** Preparation process of the HBMn-FA nanotherapeutic platform. **(b)** Mechanism of HBMn-FA-mediated cGAS-STING pathway activation for initiating systemic antitumor immunity and enhancing the therapeutic efficacy of checkpoint blockade against both localized and metastatic tumors

Hemin, buthionine sulfoximine (BSO), and Mn²⁺ were co-encapsulated into a poly(lactic-co-glycolic acid) (PLGA) matrix to synthesize HBMn nanoparticles (NPs) (Fig. 12) [146]. The PLGA surface was then further functionalized with DSPE -PEG-FA, a folate receptor-targeting stabilizer, yielding the optimized HBMn-FA nanotherapeutic platform [146]. The HBMn-FA nanoplatform drives ferroptosis in tumor cells, generating high levels of ROS that induce mitochondrial stress and release endogenous mtDNA, which synergizes with Mn²⁺ to activate the cGAS -STING pathway, while tumor-derived cytosolic dsDNA from ferroptotic cell debris further amplifies STING signaling in DCs and other antigen-presenting cells [146]. This dual activation mechanism bridges ferroptosis with innate immunity by priming robust systemic antitumor responses [146]. The amplified cGAS-STING axis enhances the efficacy of checkpoint blockade, achieving potent suppression of both localized and metastatic tumors. HBMn-FA establishes a paradigm for next -generation immunotherapies that exploit innate immune pathways for precise cancer treatment by coupling ferroptosis induction with STING pathway activation [146].

**Fig. 13:**
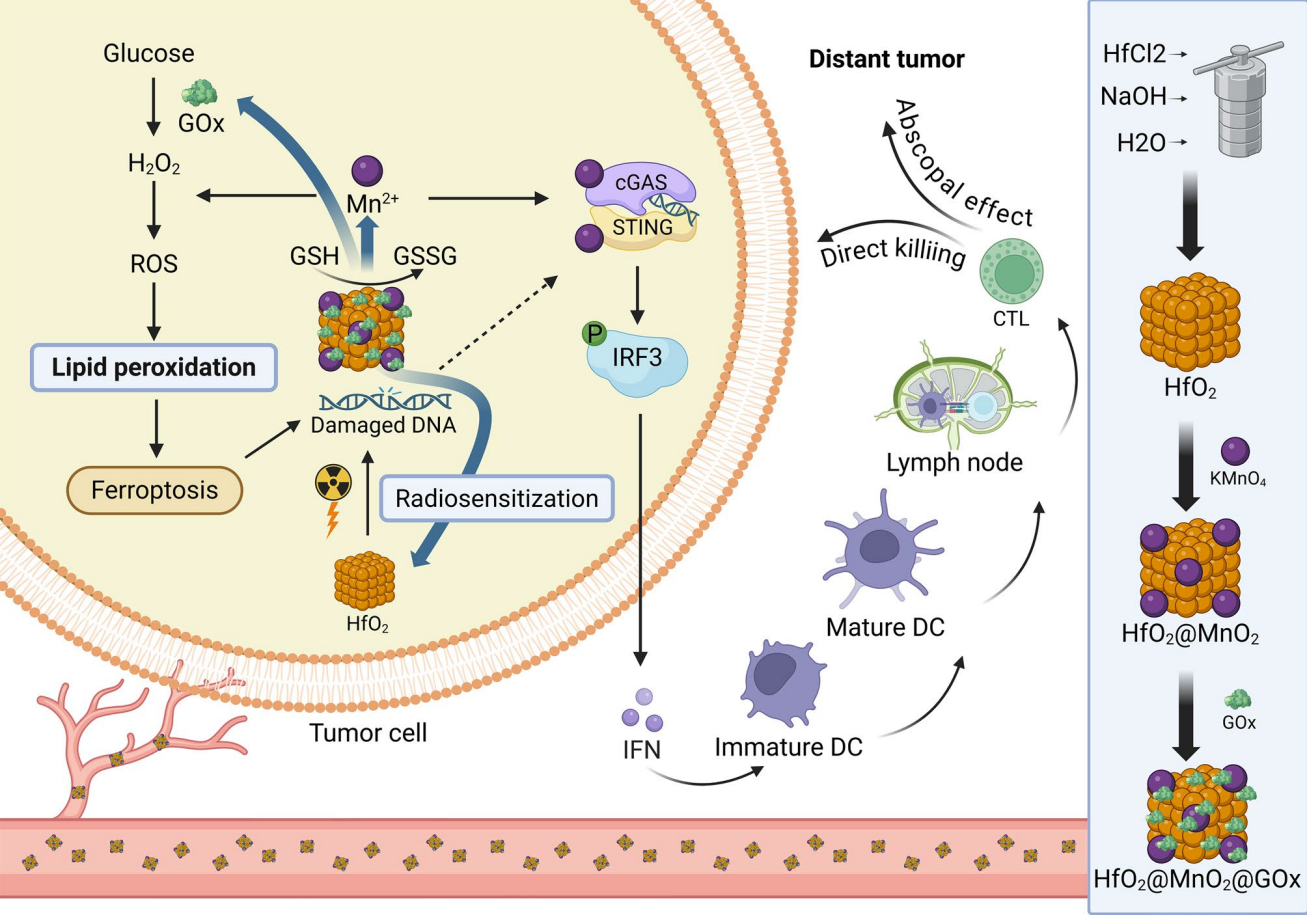
Schematic illustration of the mechanism of HfO₂@MnO₂@GOx nanoparticles in breast cancer combination therapy

Hu et al. engineered a multifunctional nanoplatform (HfO2@MnO2@GOx, HMG) by layering a manganese dioxide (MnO₂) shell onto a clinically established radiosensitizer, hafnium oxide (HfO₂), and surface-doping it with glucose oxidase (GOx) (Fig. 13) [147]. This design leverages dual ferroptosis induction via GSH depletion and ROS amplification to enhance radiotherapy efficacy [147]. The ferroptotic cascade not only intensifies DNA damage in 4T1 breast cancer cells but also synergizes with radiotherapy to activate the Mn²⁺-mediated cGAS-STING pathway, triggering robust systemic antitumor immunity [147].

This section demonstrates that diverse nanomedicine platforms (e.g., ZCUNH, DHA@MIL-101, Fe-THBQ/SR, and CMS) can induce tumor ferroptosis through metal ion-driven redox dysregulation (e.g., GSH depletion, GPX4 inhibition, and iron overload), which subsequently activates the cGAS-STING pathway through ferroptosis-derived DAMPs (e.g., mtDNA and dsDNA). This cascade remodels the immunosuppressive TME by promoting ICD, DC maturation, M1 macrophage polarization, and cytotoxic T cell infiltration, thus establishing a self-reinforcing cycle in which ferroptosis-triggered innate immunity amplifies antitumor responses. Nanocarriers exploit intrinsic properties (e.g., Mn²⁺-mediated STING potentiation, EPR effect) to spatially coordinate ferroptosis induction and immune activation, validating the ferroptosis→cGAS -STING axis as a therapeutic paradigm.

Critical unresolved questions include defining nanomaterial-specific thresholds for optimal DAMP release (e.g., mtDNA vs. nuclear DNA) that maximize STING activation without systemic inflammation and elucidating how nanocarrier composition (e.g., metal valence and surface charge) dictates crosstalk between ferroptotic damage and immune cell recruitment across tumor types. Future studies must establish standardized preclinical metrics for comparing nanoplatform efficacy (e.g., DAMP quantification and immune cell spatial mapping), develop strategies to circumvent nanoparticle-induced STING tolerance, and validate whether in vivo ferroptosis-immune synergies persist in metastatic or therapy-resistant settings through longitudinal studies. Clinical translation requires addressing metal ion biodistribution/toxicity and identifying biomarkers to stratify the patients most likely to benefit from ferroptosis-directed nanotherapies.

### Nanomedicine simultaneously activate ferroptosis and cGAS-STING

Innovative nanomedicine strategies have emerged that simultaneously co-activate ferroptosis and the cGAS-STING pathway within tumors. By engineering multifunctional nanoparticles with tumor-targeting capabilities, stimuli-responsive payload release, and synergistic component design, these platforms achieve spatiotemporally resolved dual-pathway activation through metal ions (Fe²⁺/Mn²⁺), STING agonists, or ferroptosis inducers: self-reinforcing therapeutic cycles where ferroptosis-derived DAMPs (mtDNA, HMGB1) activate cGAS-STING, while STING-driven interferon responses exacerbate oxidative stress and iron dysregulation; remodeling of immunosuppressive microenvironments through enhanced ICD, dendritic cell maturation, and cytotoxic T cell infiltration. The following section describes how rationally designed nanoplatforms leverage this dual-pathway synergy to enhance therapeutic precision, overcome resistance mechanisms, and generate systemic antitumor immunity across diverse malignancies (Table 3).

**Table 3:** Overview of nanoparticles simultaneously activate ferroptosis and cGAS-STING in cancer treatment

Nanoparticles	Loaded agent	Cancer	Tested model	Effects OR Involved mechanism	Ref
PMZFNs		Prostate cancer	PC3 cells tumor-bearing mice	PMZFNs drive dual therapeutic mechanisms by inducing ferroptosis that directly killing cancer cells while releasing tumor-associated antigens to trigger ICD and activating cGAS-STING, which amplifying ICD efficacy through interferon-β production and dendritic cell priming.	[39]
IFNγ/uMn-LDHs nanoplatform	Mn + IFNγ	Breast cancer	4T1-tumor-bearing mice	IFNγ/uMn-LDHs induces ferroptosis in cancer cell and activates the cGAS-STING pathway in TEM.Ferroptosis-induced immunogenic cell death (ICD) further liberates tumor-associated antigens, synergizing with STING-driven IFN-β production to recruit cytotoxic T lymphocytes (CTLs).	[40]
CMArg@Lip	ML385	Cholangiocarcinoma	C57BL/6 mice		[148]
REV@SR780Fe@LEV-RS17 NPs	SR780Fe + Reversine	Breast cancer	4T1-tumor-bearing mice	REV@SR780Fe@LEV-RS17 NPs synergistically combine photodynamic therapy (PDT), ferroptosis, and cGAS-STING pathway activation to enhance antitumor immunity.	[149]
MnFe₅O₈@(M1M-DOX) NPs	MnFe₅O₈+DOX	Breast cancer	4T1-tumor-bearing mice	This platform enables tumor-targeted delivery, Fe²⁺/Fe³⁺-mediated ferroptosis, and Mn²⁺-driven cGAS-STING activation.	[150]
DP-HBN/RA nanomedicine	RSL3 and diABZi	Breast cancer	4T1-tumor-bearing mice	DP-HBN/RA nanomedicine amplify radiotherapy efficacy through dual ferroptosis induction and systemic immune activation.	[151]
C5-AFt nanoparticle	Ruthenium + apoferritin	Breast cancer	4T1-tumor-bearing mice	C5-AFt NPs inducing ferroptosis via mitochondrial damage (ROS overproduction, GSH depletion, and GPX4/SLC7A11 downregulation) and activating the cGAS-STING pathway by promoting mtDNA leakage into the cytoplasm.	[152]
FeMn@cGAMP@M	Fe3O4@MnO + cGAMP	Breast cancer	4T1-tumor-bearing mice	FeMn@cGAMP@M induces dual ROS-mediated ferroptosis and cGAS-STING immune activation.	[153]
OMV/SaFeFA	Fe²⁺+agonist-4	CRC	Colon tumor-bearing mouse	Ferroptosis-derived damage-associated molecular patterns (DAMPs, e.g., calreticulin) prime DCs for antigen presentation, while STING-driven IFN-γ suppresses SLC7A11, creating a self-reinforcing loop that intensifies ferroptosis and immune activation.	[154]
NA-Ir	Iridium(III)	Melanoma	A375 cells-tumor bearing mice	Under light irradiation, NA-Ir induce ferroptosis and activates the cGAS-STING pathway, which promotes ferritinophagy and ensued ferroptosis,.	[155]
AMO	AgNO3			AMO triggers ferroptosis and pyroptosis, and concurrently activate the cGAS-STING pathway, which amplifies innate immunity.	[156]
PtMnIr	CAT, OXD, SOD, POD, GPx	Melanoma	B16-F10 melanoma-bearing C57BL/6 mice	The PtMnIr induce ferroptosis by downregulating GPX4 and promoting LPO, while simultaneously activating the cGAS-STING pathway through Mn²⁺ release, which sensitizes tumor cells to innate immune responses.	[157]
Fe-PU/CD-IPI@cBSA	IPI-549 + IPI-549	CRC		Fe-PU/CD-IPI@cBSA induced ferroptosis, activatied STING signaling pathway, and the repolarized macrophages in the mice with spontaneous tumor in the colorectal area and tumor-bearing mice.	[158]
Fe0@HMON@DNA-Exo		TNBC	4T1 tumor-bearing mice	Fe0@HMON@DNA-Exo induced ferroptosis, activatied STING signaling pathway.	[159]
AHA@MnP/QCT nanoparticles	Manganese phosphate + quercetin	NSCLC	Lewis’s cancer cell-bearing C57 mice	The AHA@MnP/QCT NPs vector sustainably releases QCT and Mn2 + into the acidic environment, which induces apoptosis and promotes ferroptosis in cells via the Fenton-like reactions. Free Mn2 + induces immunogenic cell death by activating DCs and promoting the activation and proliferation of T cells. Non-invasive imaging is achieved by accumulating AHA@MnP/QCT and enhancing T2-MRI signal at the tumor site.	[160]
MGNH nanocomplex	NLG919				[161]
FMPH	Plinabulin				

DOX, doxorubicin; MGNH, MnFe2O4@NaGdF4@NLG919@HA; NLG919, immune checkpoint indoleamine 2,3-dioxygenase (IDO) inhibitor

**Fig. 14:**
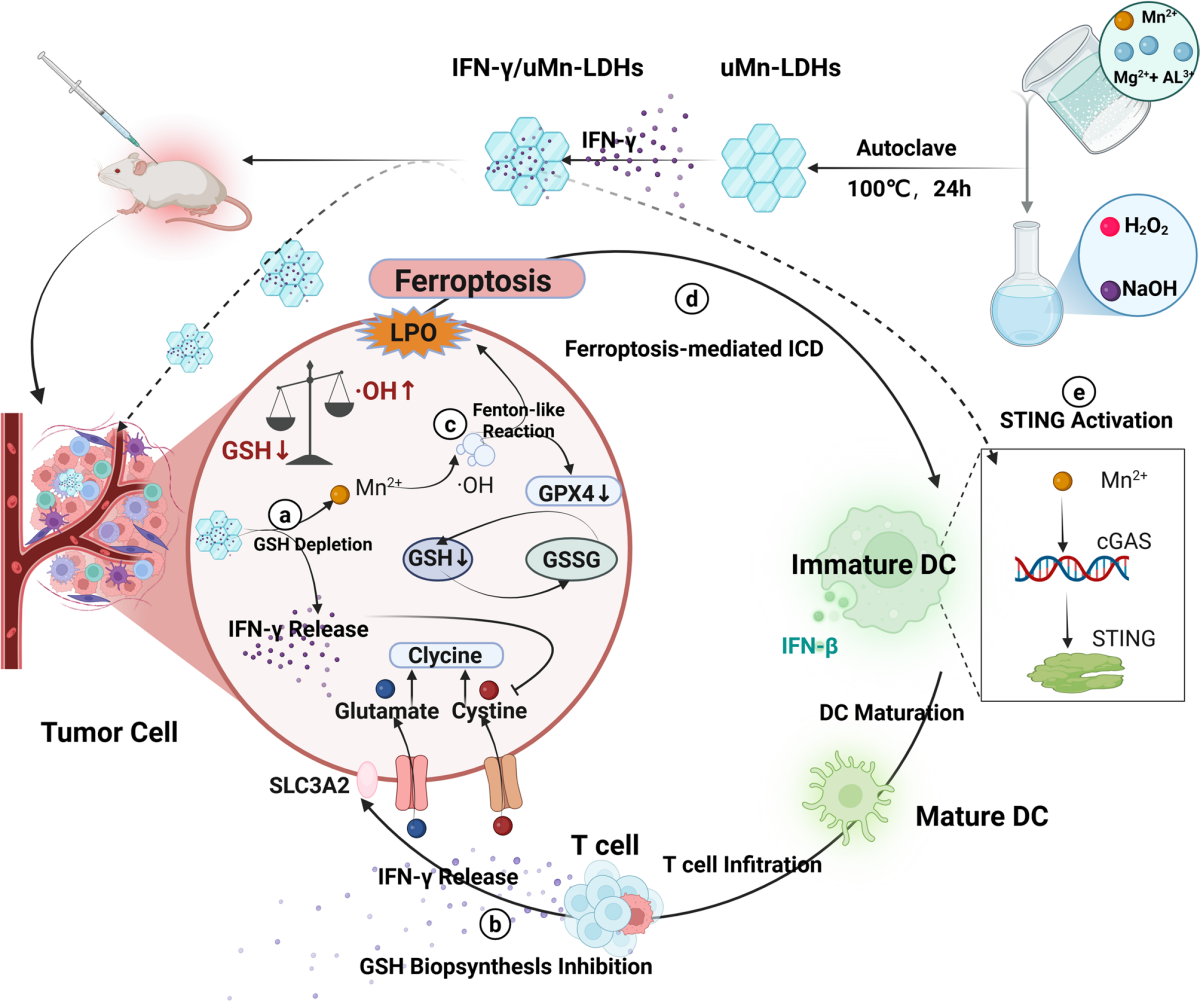
**(A)** Synthetic process of ultrathin Mn-based LDH nanosheets (uMn-LDHs) and subsequent IFN loading via electrostatic interaction. **(B)** Therapeutic mechanism of IFN/uMn-LDHs for co-enhancement of ferroptosis and antitumor immunity. Internalized IFN/uMn-LDHs consume excessive intracellular GSH and release Mn²⁺ to produce toxic ·OH via Fenton-like reactions, inducing ferroptosis. Delivered IFN downregulates membrane-localized SLC7A11 expression, inhibiting cystine uptake and subsequently interrupting GSH biosynthesis, cooperating with uMn-LDHs to enhance ferroptosis. In the TME, IFN/uMn-LDHs activate the cGAS-STING signaling pathway and stimulate DC maturation. Combined with ferroptosis-mediated ICD, cytotoxic T cell tumor infiltration is facilitated; secreted IFN cytokines further promote ferroptotic cancer cell death, achieving a “closed-loop” therapy that targets primary and distant tumors. (Abbreviations: GSH, glutathione; ·OH, hydroxyl radical; IFN, interferon; TME, tumor microenvironment; DC, dendritic cell; ICD, immunogenic cell death)

Prostate cancer therapy is hindered by inadequate drug accumulation, apoptosis resistance, and immunosuppression due to impaired ICD and cGAS-STING pathway inhibition. Magnetic nanomaterials can leverage the EPR effect under external magnetic fields, and their efficacy diminishes with distance, which is a critical limitation given the deep pelvic location of prostate tumors. Wang et al. developed PEGylated manganese-zinc ferrite nanocrystals (PMZFNs) and introduced an innovative strategy involving intratumoral implantation of micromagnets to establish a sustained internal magnetic field (Fig. 14; Table 3) [39]. This approach actively recruits intravenously administered PMZFNs, achieving highly efficient tumor accumulation and bypassing the spatial constraints of external magnets. PMZFNs drive dual therapeutic mechanisms by inducing ferroptosis that directly kills cancer cells while releasing tumor-associated antigens to trigger ICD and activating cGAS -STING, which amplify the efficacy of ICD through interferon-β production and dendritic cell priming [39]. The synergy between ferroptotic tumor ablation and STING-mediated immune activation reshapes the immunosuppressive microenvironment and fosters cytotoxic T cell infiltration [39]. In vivo, this system demonstrates potent tumor suppression with minimal systemic toxicity [39]. This study pioneered a paradigm shift from passive EPR reliance to active magnetic targeting, offering a translatable solution to enhance therapeutic precision and overcome resistance in deep-seated malignancies.

The IFN-γ/uMn-LDH nanoplatform operates through a dual-action mechanism to synergistically amplify ferroptosis and antitumor immunity [40]. Upon tumor cell internalization, IFN-γ/uMn-LDH deplete intracellular GSH through Mn²⁺-mediated Fenton-like reactions, generating cytotoxic •OH to drive ferroptosis [40]. Concurrently, IFN-γ suppresses cystine uptake by downregulating membrane -localized SLC7A11, disrupting GSH biosynthesis, and enhancing vulnerability to ferroptosis [40]. In the TME, the released Mn²⁺ activates the cGAS-STING pathway, stimulating DC maturation and antigen presentation [40]. Ferroptosis-induced ICD further liberates tumor-associated antigens and synergizes with STING-driven IFN-β production to recruit CTLs. Critically, infiltrating CTLs secrete IFN-γ, establishing a self-reinforcing cycle that enhances ferroptosis while sustaining immune activation [40]. This “closed-loop” strategy not only eradicates primary tumors but also induces systemic immunity to suppress distant metastases, overcoming the traditional limitations of monotherapeutic approaches [40].

Hu et al. developed an innovative nanoplatform (CMArg@Lip) that synergistically integrates photodynamic therapy, nitric oxide (NO)-mediated gas therapy, and NRF2 pathway inhibition through ML385 [148]. This system overcomes TME limitations by concurrently generating cytotoxic reactive species (ROS/RNS including ONOO⁻) through Ce6 photosensitization and l-arginine-derived NO production, while ML385 disrupts NRF2-mediated antioxidant defenses (GSH/GPX4 axis) to amplify oxidative stress [148]. In cholangiocarcinoma models, CMArg@Lip induced ferroptosis through GPX4/GSH depletion and triggered mitochondrial DNA release from myeloid-derived suppressor cells (MDSCs), thereby activating the cGAS-STING pathway [148]. This dual mechanism achieved reversal of immunosuppression through reprogramming of MDSCs to pro-immunogenic phenotypes, enhanced CTL activity through PD-L1 downregulation, amplified ICD via ferroptosis-mediated DAMP release (HMGB1, ATP), and dendritic cell maturation and T cell infiltration potentiation. The platform establishes a self -reinforcing therapeutic cycle where STING activation and ferroptosis-derived immunostimulation cooperatively remodel the TME, creating sustained antitumor immunity [148].

**Fig. 15:**
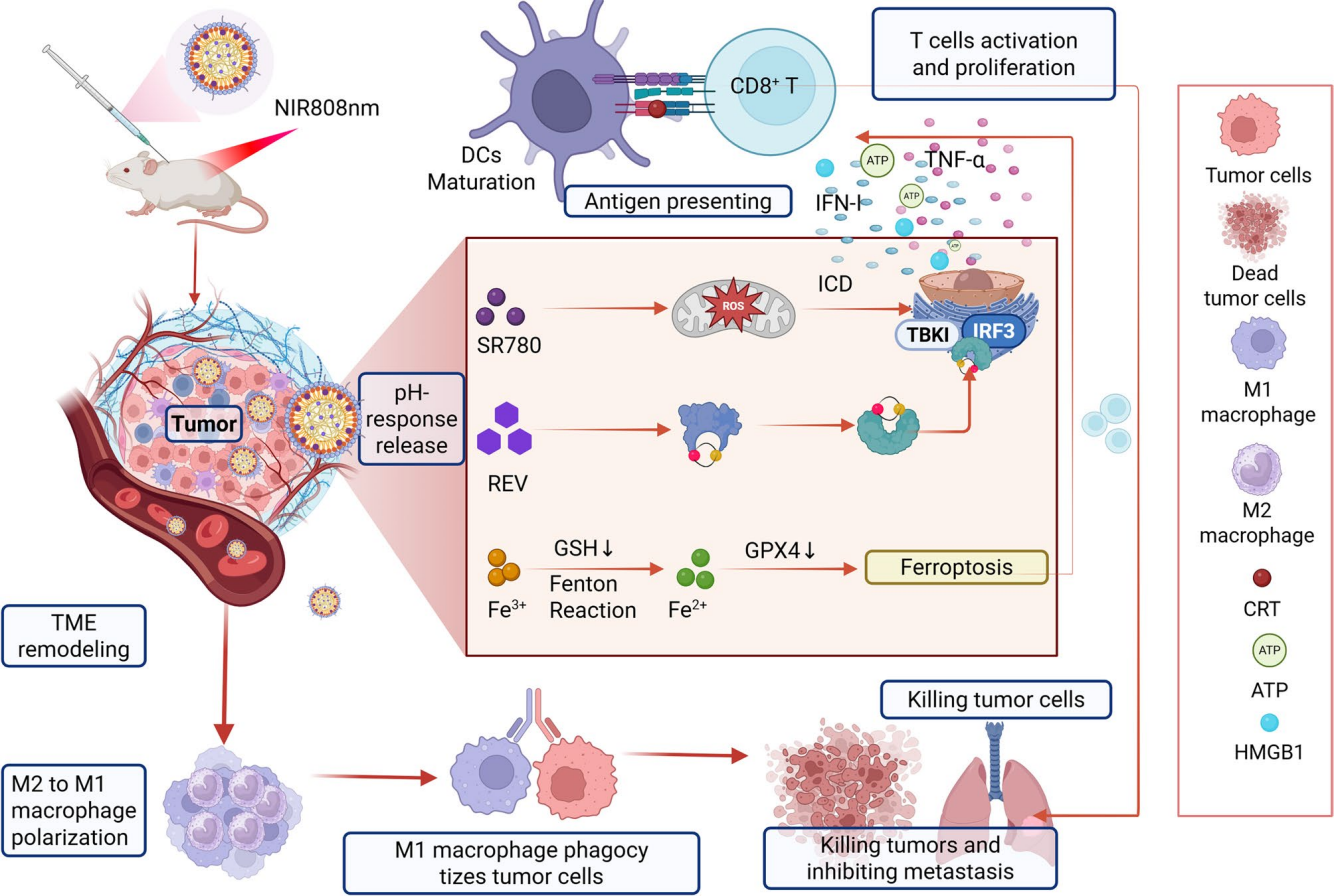
Illustration of REV@SR780Fe@LEV-RS17 NPs and their antitumor mechanisms. **(a)** Fabrication of REV@SR780Fe@LEV-RS17 NPs. **(b)** Structure conversion of SR780 and SR780Fe in Fe³⁺-rich and acidic environments, respectively. **(c)** SR780Fe activation in the TME: Released SR780 produces ROS under 808 nm laser irradiation; released Fe³⁺ ions are reduced to Fe²⁺ by GSH, inducing ferroptosis; REV activates the cGAS-STING pathway to enhance innate immunity

pH-activatable hybrid nanovesicles (REV@SR780Fe@LEV-RS17 NPs) derived from M1 macrophage extracellular vesicles (EVs) and liposomes have been developed in another study (Fig. 15) [149]. These nanoparticles synergistically combined PDT, ferroptosis, and cGAS-STING pathway activation to enhance antitumor immunity [149]. An acidic TME triggers the release of SR780 (a photosensitizer) and Fe^3+^, which are reduced to Fe^2+^ by GSH, inducing LPO and ferroptosis, which generates ROS to cause mtDNA leakage and ICD, releasing DAMPs (e.g., ATP, HMGB1, and CRT) to activate DCs [149]. Reversine (REV), a cGAS-STING agonist, activates innate immunity, promoting the production of cytokines (e.g., IFN-β) and T cell infiltration. Triple therapy inhibits primary tumor growth, metastasis (lung/liver), and recurrence in 4T1 breast cancer models while reprogramming the immunosuppressive TME through M1 macrophage polarization [149].

**Fig. 16:**
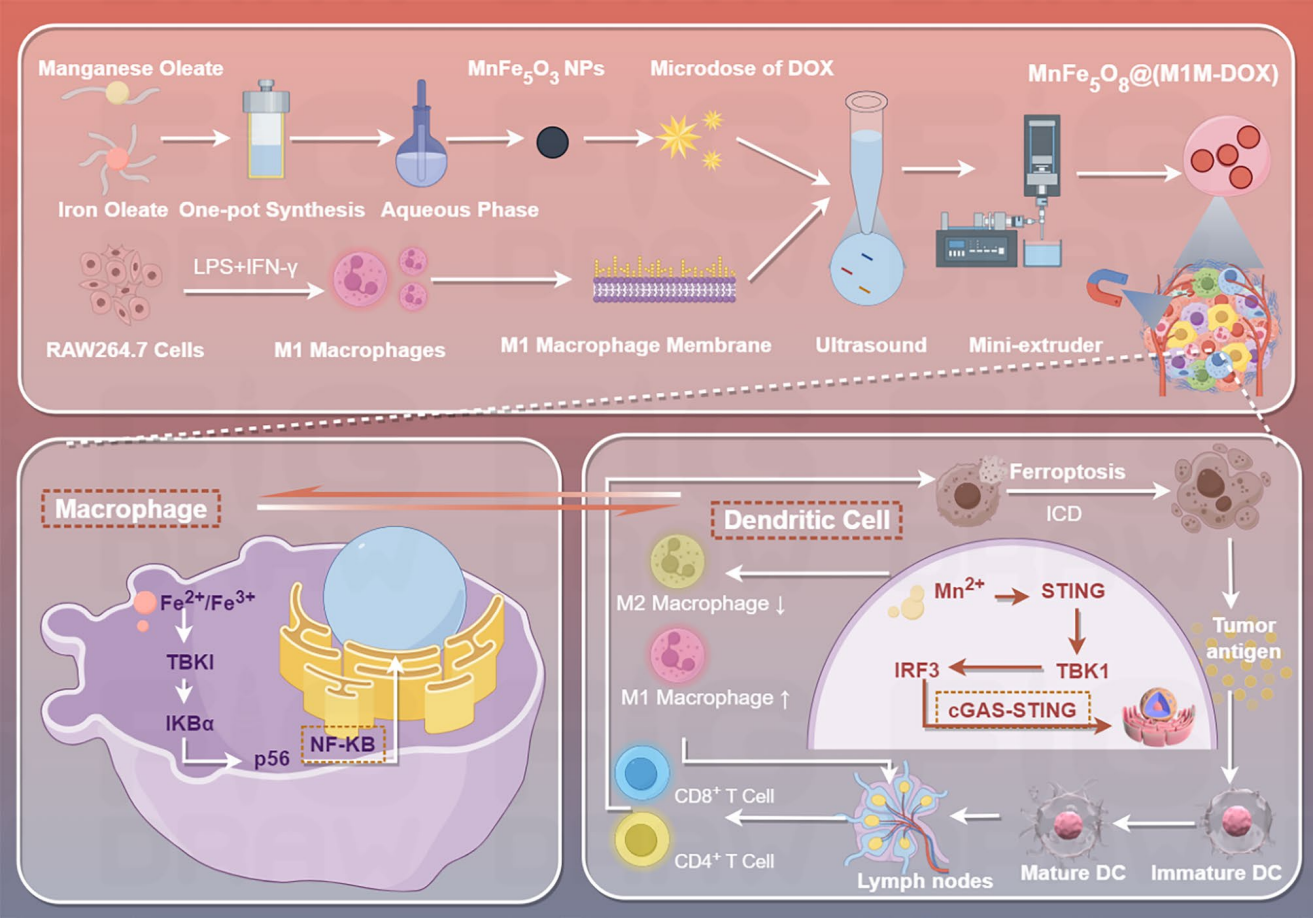
Schematic Illustration of MnFe₅O₈@(M1M-DOX) NPs Preventing Primary Tumor Progression and Recurrence. Fabrication of MnFe₅O₈@(M1M-DOX) NPs: MnFe₅O₈ and DOX are encased within the M1 macrophage membrane (M1M) via ultrasonication and mechanical extrusion. Regulatory mechanism in the TME: Fe²⁺/Fe³⁺ released by MnFe₅O₈@(M1M-DOX) drives TAMs into an M1-like state via the NF-κB pathway, promotes STING activation, enhances macrophage maturation, and primes T cells into active subtypes. Concurrently, Fe²⁺/Fe³⁺ and DOX induce tumoral ferroptosis, exposing endogenous tumor antigens. Mn²⁺/Mn³⁺ promotes DC activation via the cGAS-STING pathway. STING activation triggers downstream NF-κB signaling, inducing a pronounced pro-inflammatory cytokine response

Chen et al. developed MnFe₅O₈@(M1M-DOX) NPs, integrating manganese ferrite (MnFe₅O₈), low-dose doxorubicin (DOX), and M1 macrophage membranes (M1M) (Fig. 16) [150]. This platform enables tumor-targeted delivery, Fe²⁺/Fe³⁺ -mediated ferroptosis, and Mn²⁺-driven cGAS-STING activation. Fe²⁺/Fe³⁺ and DOX synergistically triggered tumor ferroptosis through LPO and GPX4 suppression, releasing DAMPs (e.g., mtDNA, HMGB1) to activate ICD [150]. Fe²⁺/Fe³⁺ promoted M2-to-M1 macrophage repolarization via NF-κB signaling, enhancing antitumor cytokine secretion [150]. Mn²⁺ amplified cGAS-STING signaling in dendritic cells (DCs), boosting IFN-β production and antigen presentation. Coactivation of NF-κB and cGAS-STING pathways induced robust DC maturation, TAM reprogramming, and cytotoxic T cell infiltration, suppressing primary tumor growth and recurrence in 4T1 breast cancer models [150].

**Fig. 17:**
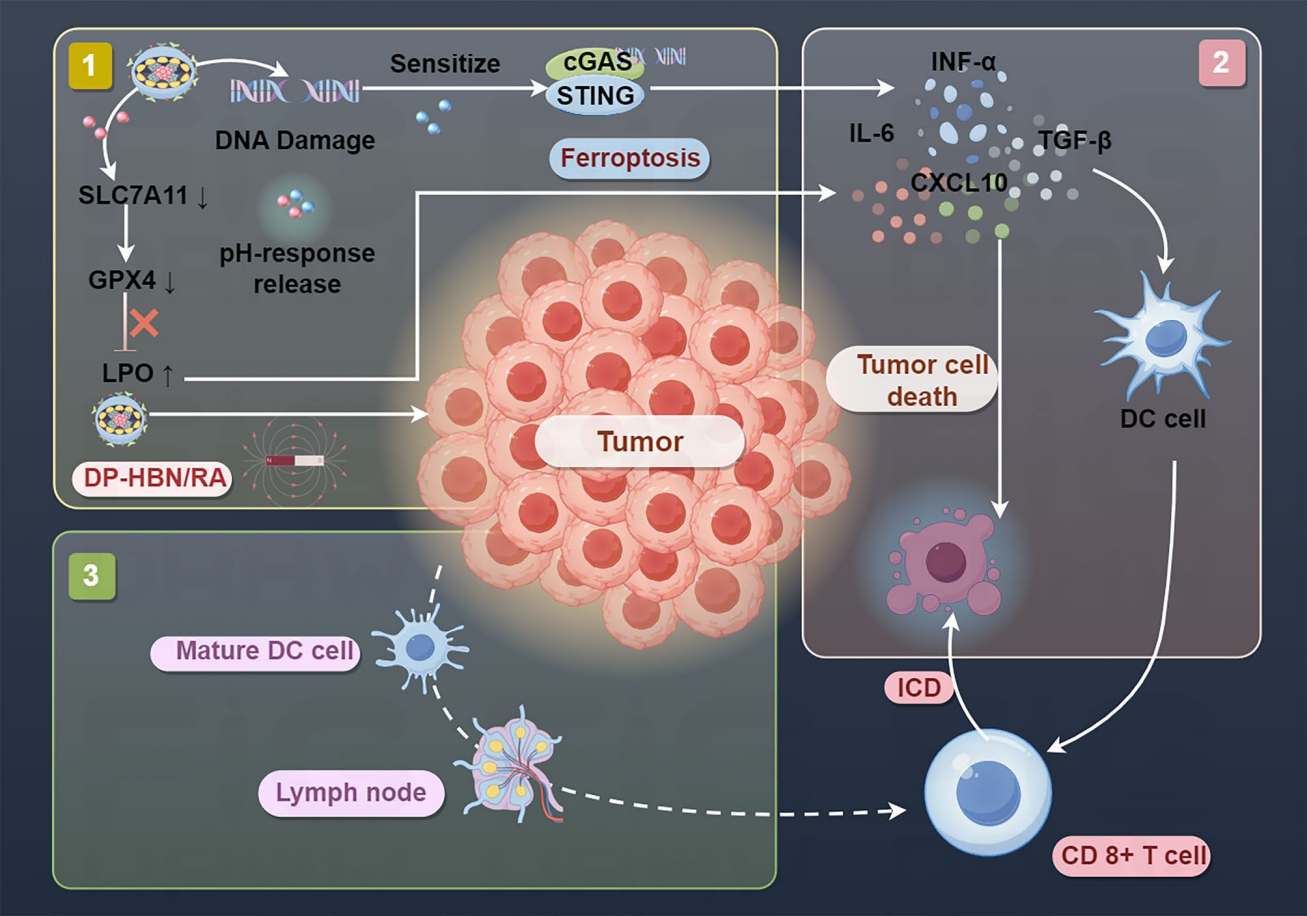
Schematic illustration of the therapeutic mechanism of DP-HBN/RA with concurrent amplification of ferroptosis and immune system activation

Aishajiang et al. developed pH-responsive DP-HBN/RA, a TME-responsive nanoplatform integrating hollow Bi2Se3 with RSL3 and diABZi to amplify the efficacy of radiotherapy through dual ferroptosis induction and systemic immune activation (Fig. 17) [151]. The Bi2Se3 core enhances X-ray energy deposition, generating tumor-localized ROS bursts that drive LPO-mediated ferroptosis. Acid-triggered RSL3 release in tumors disrupts GPX4 activity and intensifies ferroptotic cell death [151]. Radiation-induced DNA fragmentation activates cGAS -STING signaling, which is synergistically enhanced by diABZi to promote dendritic cell maturation and T cell infiltration and sustain systemic antitumor immunity. Ferroptosis-derived DAMPs and STING-mediated immune activation create a feed-forward loop for TME remodeling [151].

**Fig. 18:**
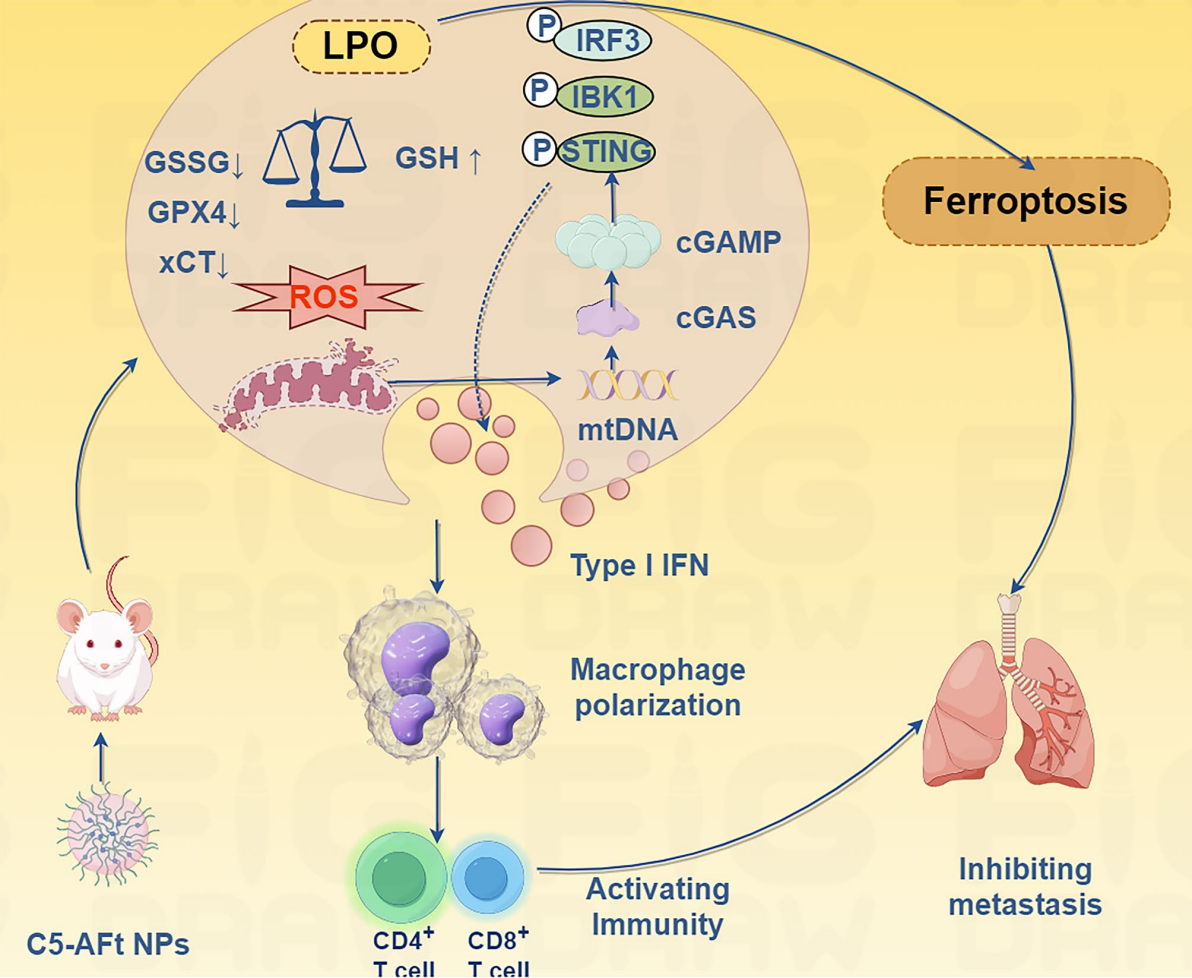
Mechanism of C5 inhibiting TNBC growth and metastasis through integrated chemotherapy and immunotherapy

A delivery system using binuclear ruthenium(II) complex (C5) and its apoferritin nanoparticle (C5-AFt NPs) was developed to exert potent antitumor effects against TNBC (Fig. 18) [152]. C5 selectively accumulates in the mitochondria, inducing ferroptosis via mitochondrial damage (ROS overproduction, GSH depletion, and GPX4/SLC7A11 downregulation) and activating the cGAS-STING pathway by promoting mtDNA leakage into the cytoplasm [152]. C5-AFt NPs enhanced tumor targeting, reduced systemic toxicity, and improved therapeutic outcomes in vivo [152].

**Fig. 19:**
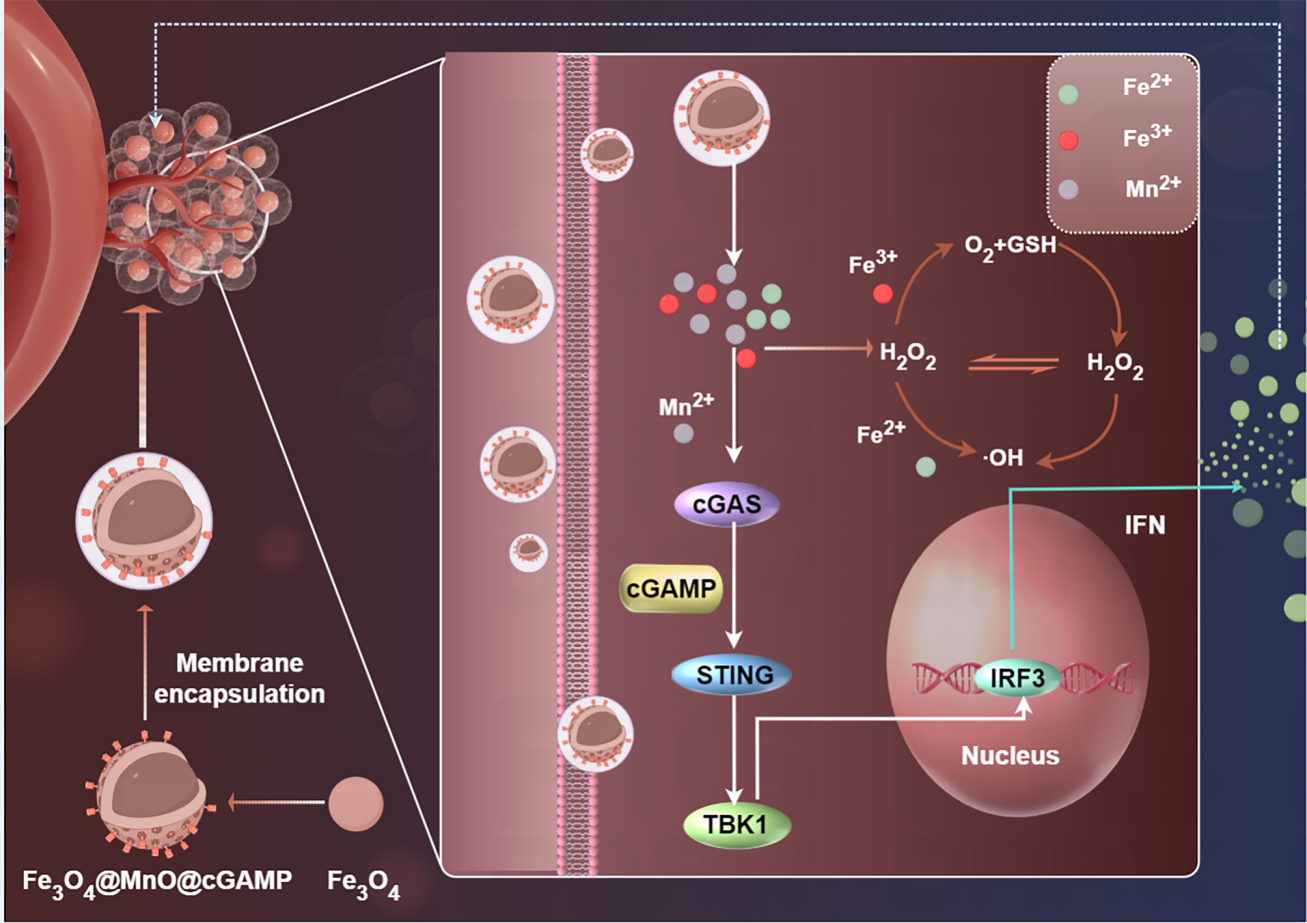
Illustration of the preparation of membrane-cloaked FeMn@cGAMP and its cancer therapy mechanism

Chai et al. developed engineered tumor membrane-coated Fe3O4@MnO @cGAMP nanoparticles (FeMn@cGAMP@M) that were used for dual ROS -mediated ferroptosis and cGAS-STING immune activation (Fig. 19) [153]. Fe^3+^/Fe^2+^ redox cycling depletes GSH and generates •OH via Fenton reactions, inducing LPO and ferroptosis [153]. Acid-triggered Mn^2+^ and cGAMP release synergistically activate the cGAS-STING pathway, stimulating macrophage recruitment, secretion of pro-inflammatory cytokines (e.g., TNF-α, IFN-β), and T/B cell infiltration [153]. The homologous membrane coating enhances tumor specificity, prolongs circulation, and protects cGAMP from degradation, achieving 76% tumor suppression in vivo with minimal systemic toxicity. This study established a dual-action paradigm where ferroptosis and cGAS-STING activation mutually potentiate oxidative damage and immune activation, offering a novel strategy for refractory cancer therapy [153].

**Fig. 20:**
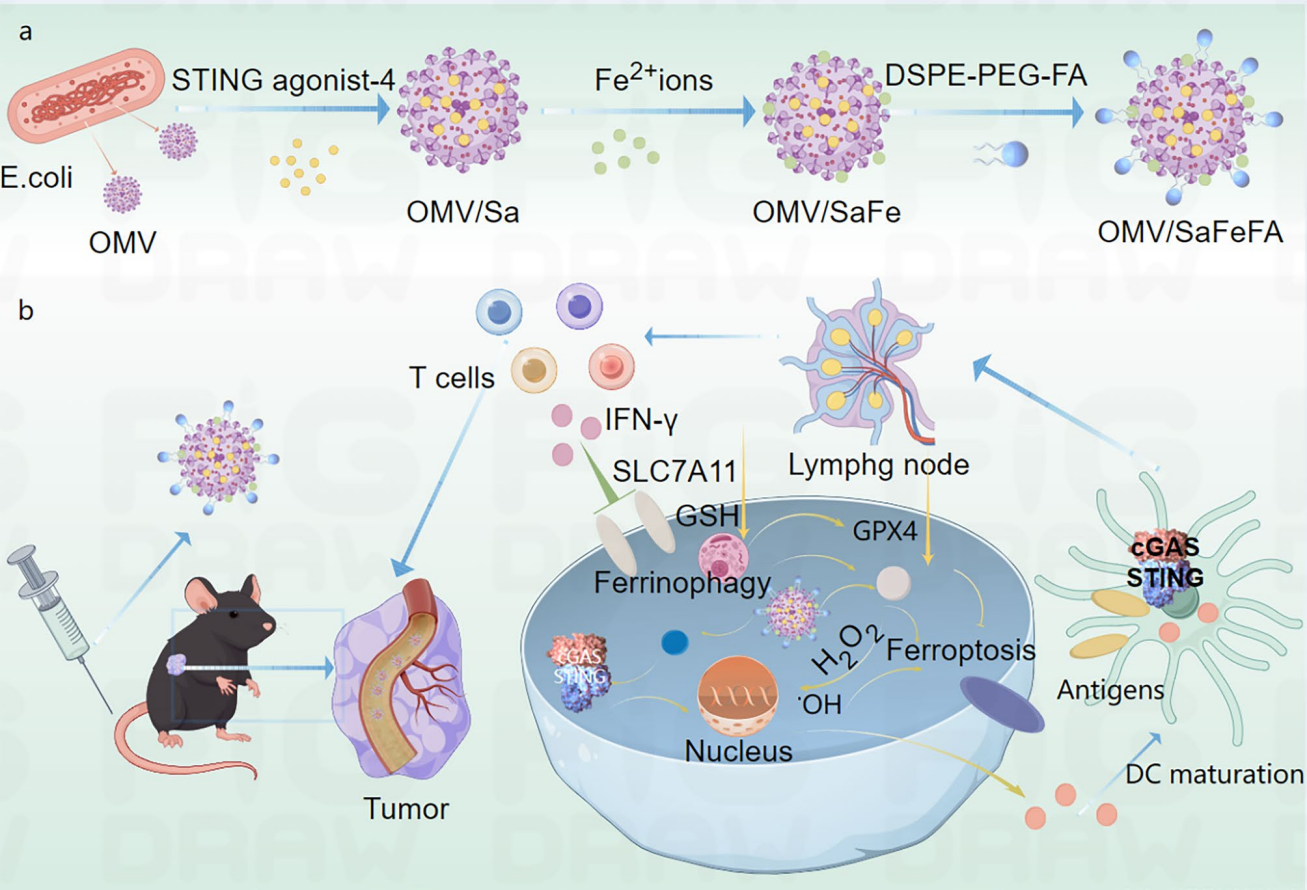
**(a)** Preparation process of OMV/SaFeFA using engineered outer membrane vesicles (OMVs) derived from Escherichia coli. **(b)** Schematic illustration of OMV/SaFeFA for tumor ferroptosis activation and antitumor immune response elicitation

Sun et al. developed a tumor-targeted nanoplatform (OMV/SaFeFA) by engineering bacterial outer membrane vesicles (OMVs) with Fe²⁺ ions, a STING agonist (agonist-4), and folate receptor-targeting DSPE-PEG-FA [154] (Fig. 20). This system synergistically induces ferroptosis and activates the cGAS-STING pathway to enhance the antitumor efficacy. Fe²⁺ catalyzes H₂O₂ into •OH, triggering LPO and depleting GSH by inhibition of SLC7A11/GPX4, thereby amplifying oxidative damage [154]. Released agonist-4 activates cGAS-STING signaling in dendritic cells (DCs), promoting IFN-β secretion and DC maturation, which enhances T cell infiltration (CD8⁺/CD4⁺) and systemic antitumor immunity [154]. Ferroptosis -derived DAMPs, e.g., calreticulin, prime DCs for antigen presentation, while STING-driven IFN-γ suppresses SLC7A11, creating a self-reinforcing loop that intensifies ferroptosis and immune activation [154]. OMV/SaFeFA achieved 77.6% tumor weight inhibition in colon cancer models with minimal toxicity, demonstrating biocompatibility and translational potential. Together, the integration of Fe²⁺-driven ferroptosis and STING agonist-triggered immunotherapy in OMV/SaFeFA represents a potent strategy to synergize oxidative damage and immune activation, offering a biocompatible and effective approach for refractory cancer therapy [154].

**Fig. 21:**
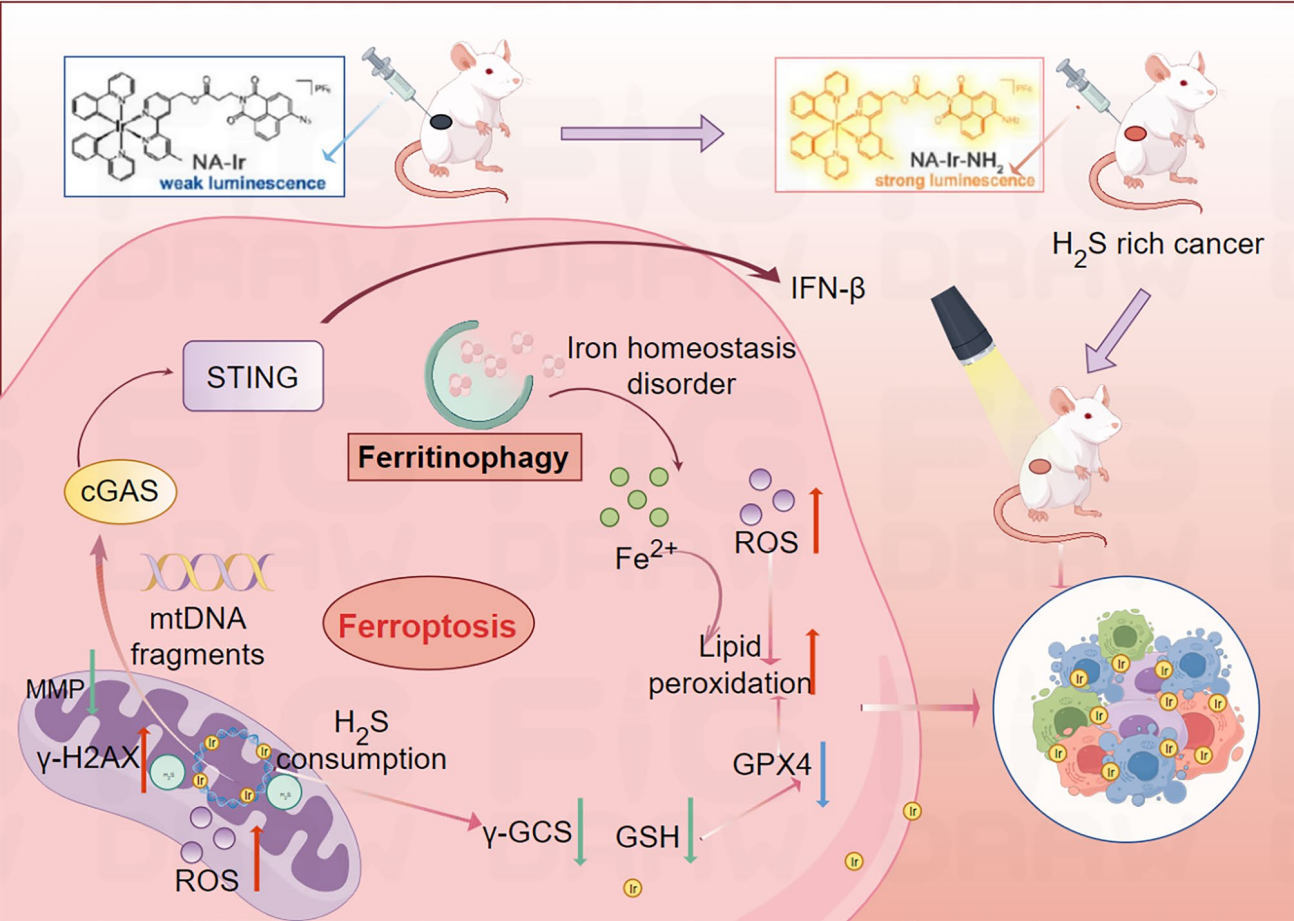
Schematic Illustration of the Mechanisms of Cancer-Specific Complex NA-Ir: Selective Lighting of H₂S-Rich Cancer Cells, Mitochondrial Localization, Mitochondrial Function Disruption, and Ultimate Induction of LPO-Mediated Ferroptosis through Different Pathways under Light Irradiation

Li et al. designed an H_2_S-responsive iridium(III) complex, NA-Ir, which selectively targets H_2_S-rich cancer cells, localizes to the mitochondria, and induces ferroptosis via photodynamic therapy (PDT) (Fig. 21) [155]. Under light irradiation, NA-Ir generates ROS, depletes GSH, and downregulates GPX4, triggering LPO, a hallmark of ferroptosis. Crucially, the mtDNA damage caused by NA-Ir activates the cGAS-STING pathway, which links DNA damage sensing to ferroptosis amplification. Activation of cGAS-STING promotes ferritinophagy (autophagic degradation of ferritin) and releases stored iron into the cytosol. Iron overload exacerbates LPO and ferroptosis, and STING-driven autophagy further enhances these processes [155]. Thus, cGAS-STING activation acts synergistically with ferroptosis by modulating iron homeostasis and amplifying oxidative stress, establishing a novel therapeutic axis for cancer-specific therapies [155]. This study pioneered the integration of cGAS-STING activation and ferroptosis induction, offering a dual-pathway strategy to overcome the limitations of monotherapy [155].

**Fig. 22:**
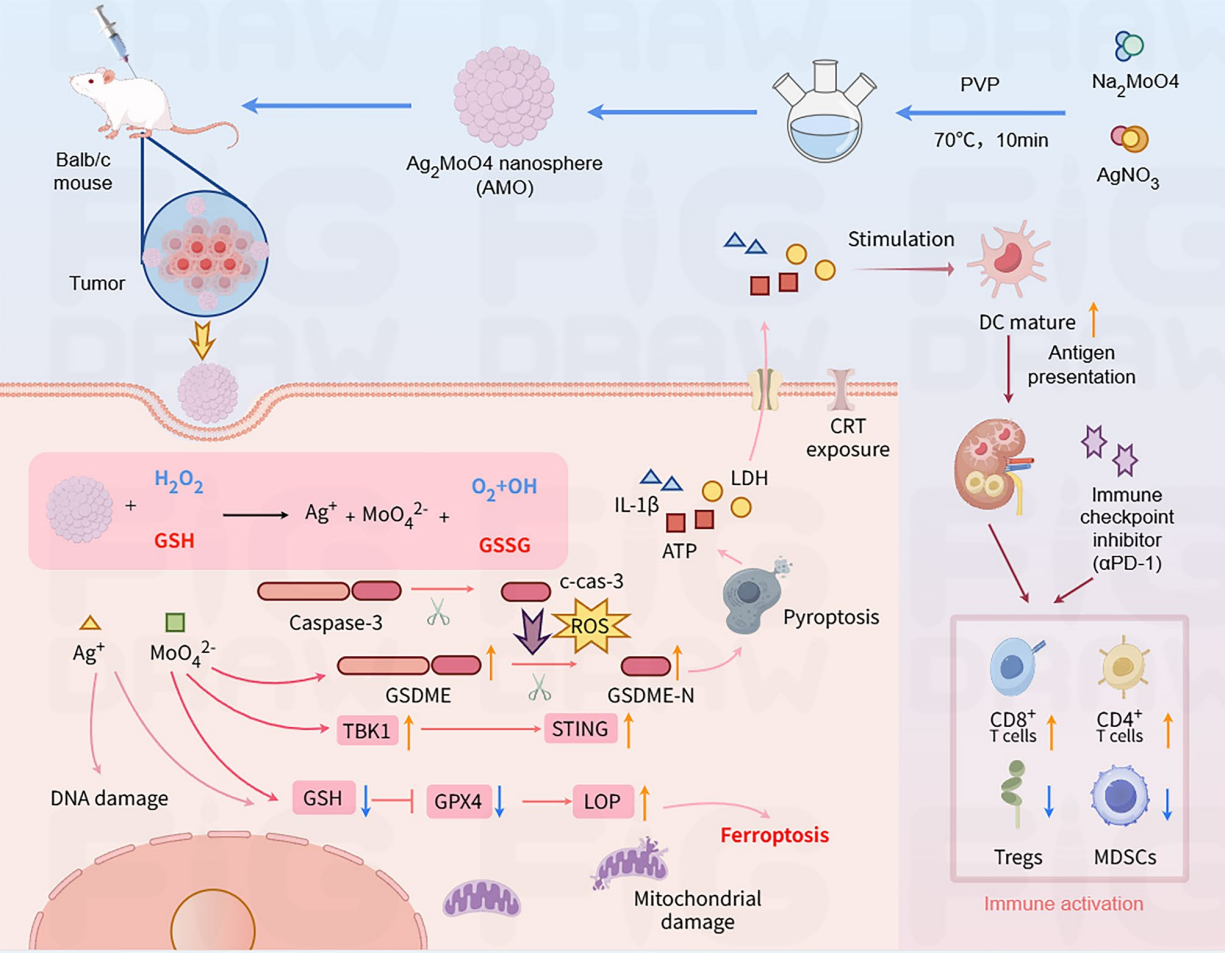
Schematic illustration of the synthesis, anti-tumor effect, and immune activation of AMO. AMO is synthesized via a rapid solvothermal method. Upon entering tumor cells, AMO consumes GSH, degrades H₂O₂ to generate ROS, and releases MoO₄²⁻ and Ag⁺. Intracellular GSH depletion and ROS induction lead to ferroptosis. Additionally, MoO₄²⁻ upregulates intracellular GSDME, and combined with ROS and Ag⁺ stimuli, cells undergo caspase-3/GSDME-mediated pyroptosis. Ferroptosis and pyroptosis synergistically enhance the immune response

Chen et al. synthesized silver molybdate nanoparticles (AMO) that induced pyroptosis and ferroptosis to enhance antitumor immunity (Fig. 22) [156]. AMO responds to TME cues (H_2_O_2_ and GSH) to release Ag^++^ and MoO_4_^2−^ ions, generating ROS and depleting GSH. This triggers ferroptosis via downregulation of LPO and GPX4. MoO_4_^2−^ uniquely upregulates GSDME expression, redirecting caspase-3 -mediated apoptosis to pyroptosis and enhancing immunogenicity. Concurrently, ROS and DNA damage activate the cGAS-STING pathway, which amplifies innate immunity by promoting type I interferon production and immune cell recruitment [156].

**Fig. 23:**
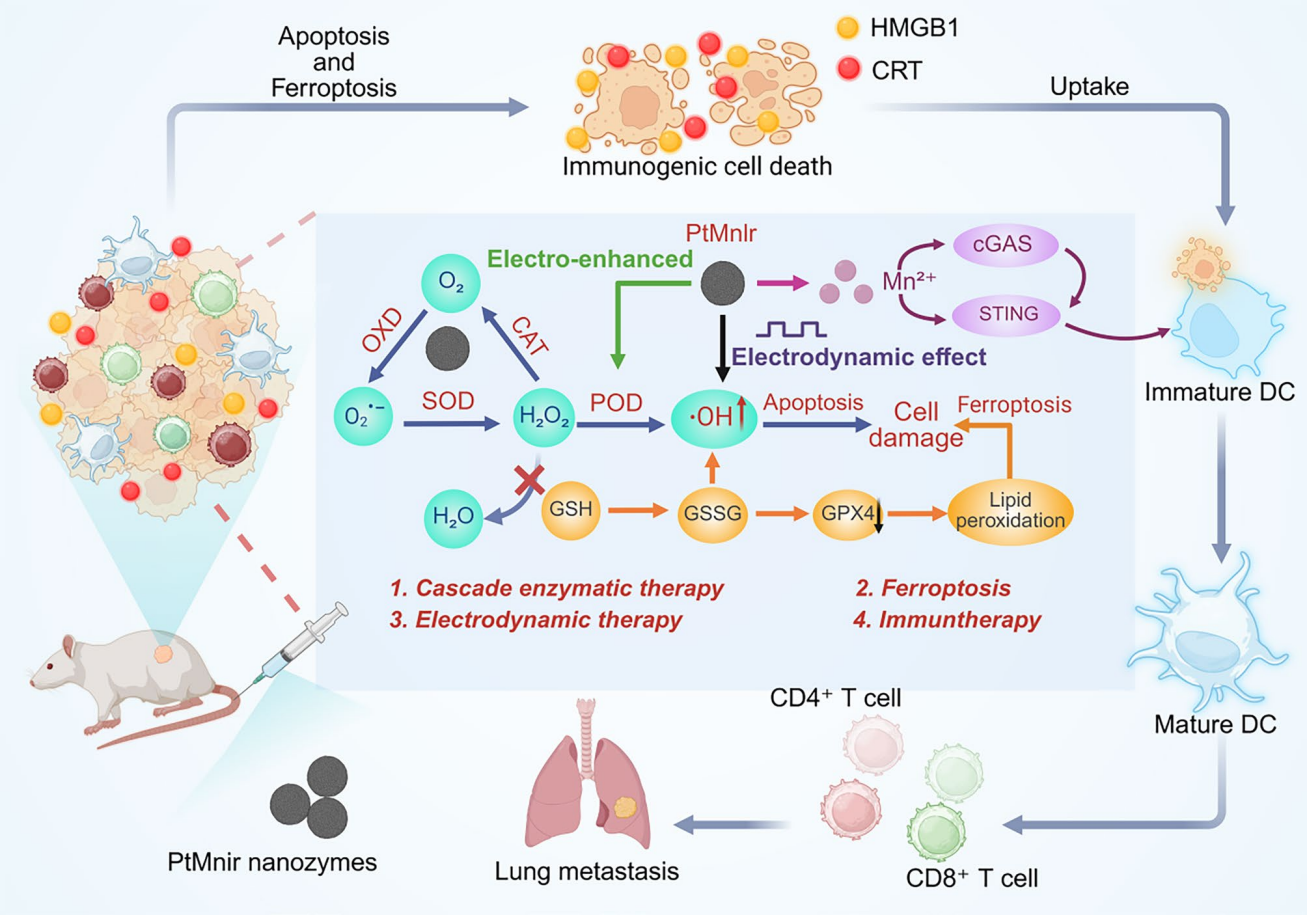
Schematic diagram of the antitumor mechanism of PtMnIr nanozymes

The study demonstrated the development of PtMnIr nanozymes, which exhibit multienzymatic activities (CAT, OXD, SOD, POD, GPx) to generate ROS and deplete GSH in a self-sustaining “inner catalytic loop,” synergized with electrodynamic therapy (EDT) under alternating current (Fig. 23) [157]. The PtMnIr induce ferroptosis by downregulating GPX4 and promoting LPO, while simultaneously activating the cGAS-STING pathway through Mn²⁺ release, which sensitizes tumor cells to innate immune responses [157]. The interplay between ferroptosis and cGAS-STING is pivotal: ferroptosis-driven ICD releases DAMPs (e.g., HMGB1, CRT), while Mn²⁺ amplifies cGAS-STING signaling to enhance type I interferon production, collectively inhibiting tumor growth and metastasis. PtMnIr suppressed primary tumor progression and almost completely eradicated lung metastases in the combined EDT/nanozyme treatment of B16-F10 melanoma-bearing C57BL/6 mice, underscoring the dual ferroptosis-immunotherapy mechanism [157].

**Fig. 24:**
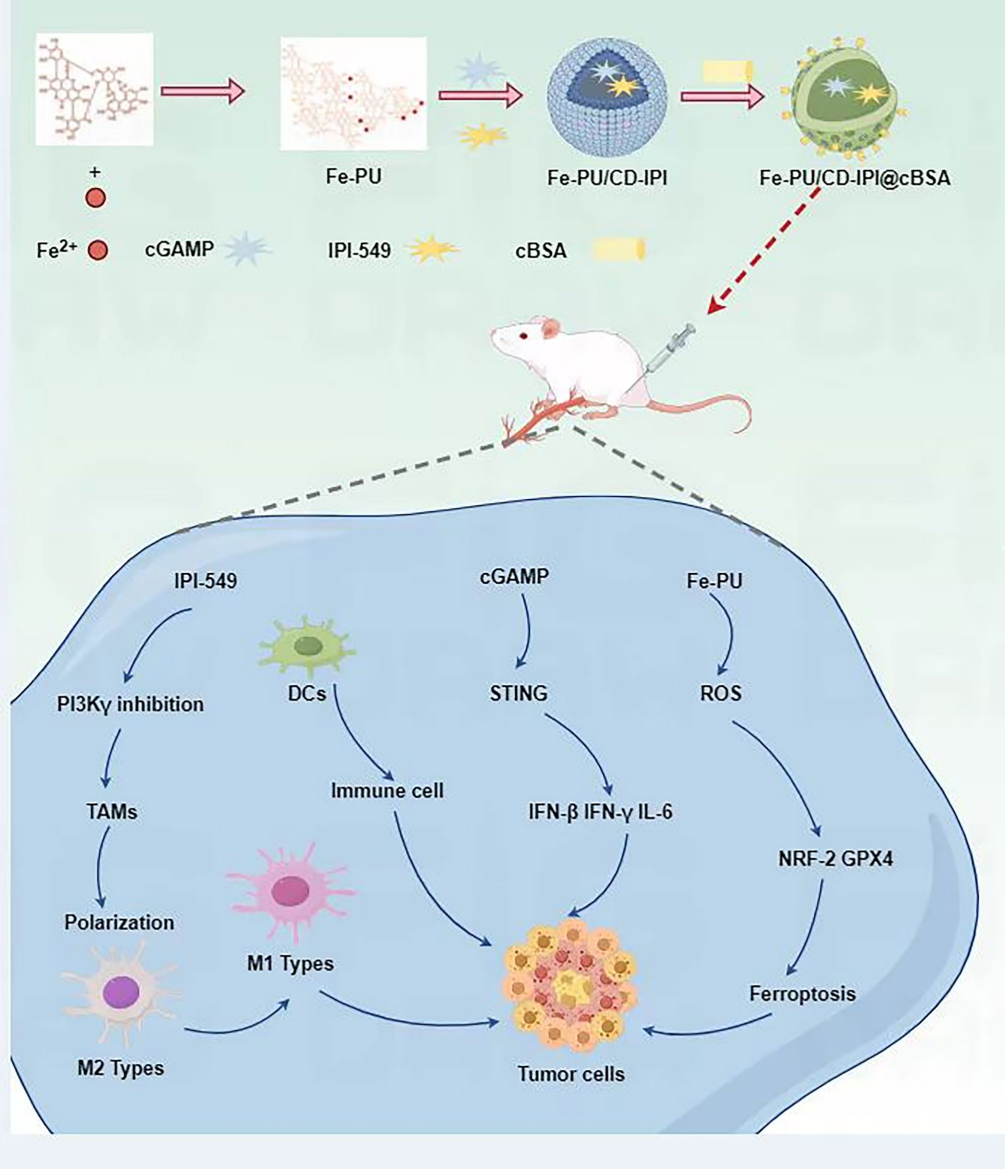
The synthetic process of Fe-PU/CD-IPI@cBSA and its comprehensive cancer therapy mechanism

Zheng et al. developed a ferric ion-punicalagin coordination network (Fe-PU) that functions as a ferroptosis inducer and a nanocarrier for co-loading cGAMP and IPI-549, yielding the nanocomposite Fe-PU/CD-IPI (Fig. 24) [158]. To enhance tumor-targeted delivery, Fe-PU/CD-IPI was further modified with cyclic arginine -glycine-aspartic acid (cRGD) peptide-conjugated bovine serum albumin (cRGD -BSA) to generate the optimized formulation, Fe-PU/CD-IPI@cBSA [158]. Fe-PU/CD -IPI@cBSA induced ferroptosis, activated the STING signaling pathway, and repolarized macrophages in mice with spontaneous tumors in the colorectal area and in tumor-bearing mice [158]. Mechanistically, cGAMP and IPI-549 synergistically remodeled the immunosuppressive TME by amplifying ICD and inhibiting PI3K-γ-mediated immune evasion [158]. The nanohybrid system exhibited potent antitumor efficacy in in vitro models, patient-derived organoids, and in vivo settings, highlighting its translational potential for colorectal cancer therapy through coordinated ferroptosis induction and immune modulation [158].

Guo et al. presented a GSH-responsive nanoplatform, Fe0@HMON@DNA -Exo, constructed through in situ synthesis of elemental iron (Fe0) within degradable hollow mesoporous organosilica nanoparticles (HMON) followed by encapsulation with engineered EVs (DNA-Exo) derived from SN38-treated 4T1 breast cancer cells [159]. The DNA-Exo membrane confers tumor-targeting specificity through inherent homing mechanisms, thereby enabling precise delivery to TNBC cells. The system employs a dual biological logic-gate framework to orchestrate spatially controlled therapeutic responses, and OR-gated activation of the STING pathway in antigen-presenting cells (APCs) triggers robust immunostimulation, whereas an AND-gated mechanism selectively initiates Fe0 -mediated ferroptosis in TNBC cells contingent upon concurrent ROS accumulation [159]. This combinatorial logic achieved conditional payload release, simultaneously generating immunogenic ferroptosis and activating antitumor immunity [159].

**Fig. 25:**
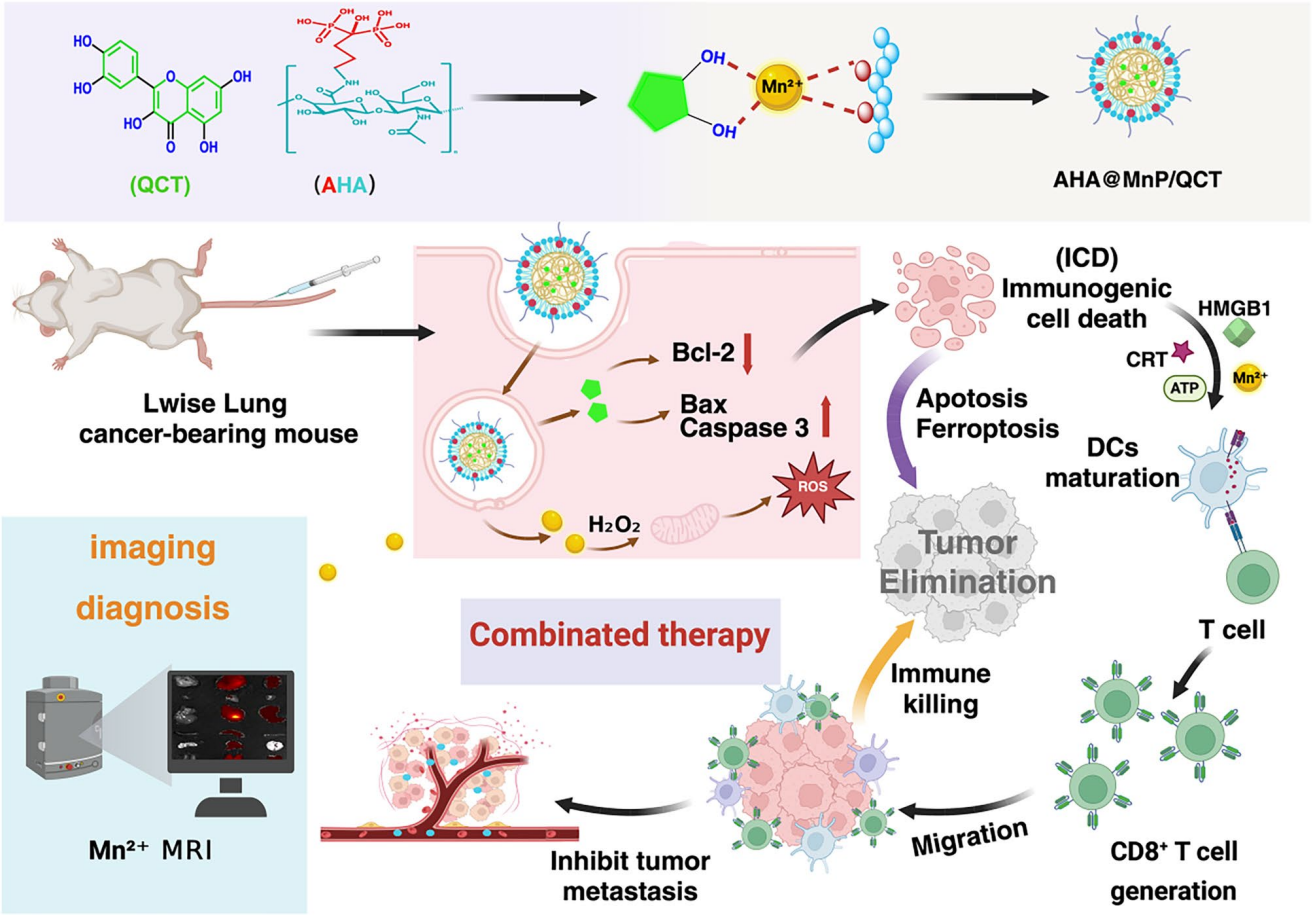
Schematic of AHA@MnP/QCT nanoparticle preparation and its one-stone-for-two-birds strategy for integrated diagnosis and treatment of non-small cell lung cancer. The AHA@MnP/QCT NP vector sustainably releases QCT and Mn²⁺ in the acidic tumor environment, inducing apoptosis and promoting ferroptosis via Fenton-like reactions. Free Mn²⁺ induces immunogenic cell death by activating DCs and promoting T cell activation and proliferation. Non-invasive imaging is achieved through tumor accumulation of AHA@MnP/QCT, enhancing the T₂-MRI signal at the tumor site

A pH-responsive core-shell nanoplatform of AHA@MnP/QCT NPs was developed. This multifunctional system leverages quercetin (QCT) and Mn²⁺ to establish a dual-modality mechanism, i.e., induction of ferroptotic cell death and activation of the cGAS-STING axis to stimulate dendritic cell maturation and CTL infiltration, orchestrating ICD while establishing antitumor immune memory (Fig. 25) [160]. The demonstrated tumor-selective action coupled with immune microenvironment remodeling positions the AHA@MnP/QCT NPs as a transformative paradigm in precision oncology, offering a clinically translatable solution to bridge diagnostic imaging and personalized therapy in NSCLC treatment [160].

**Fig. 26:**
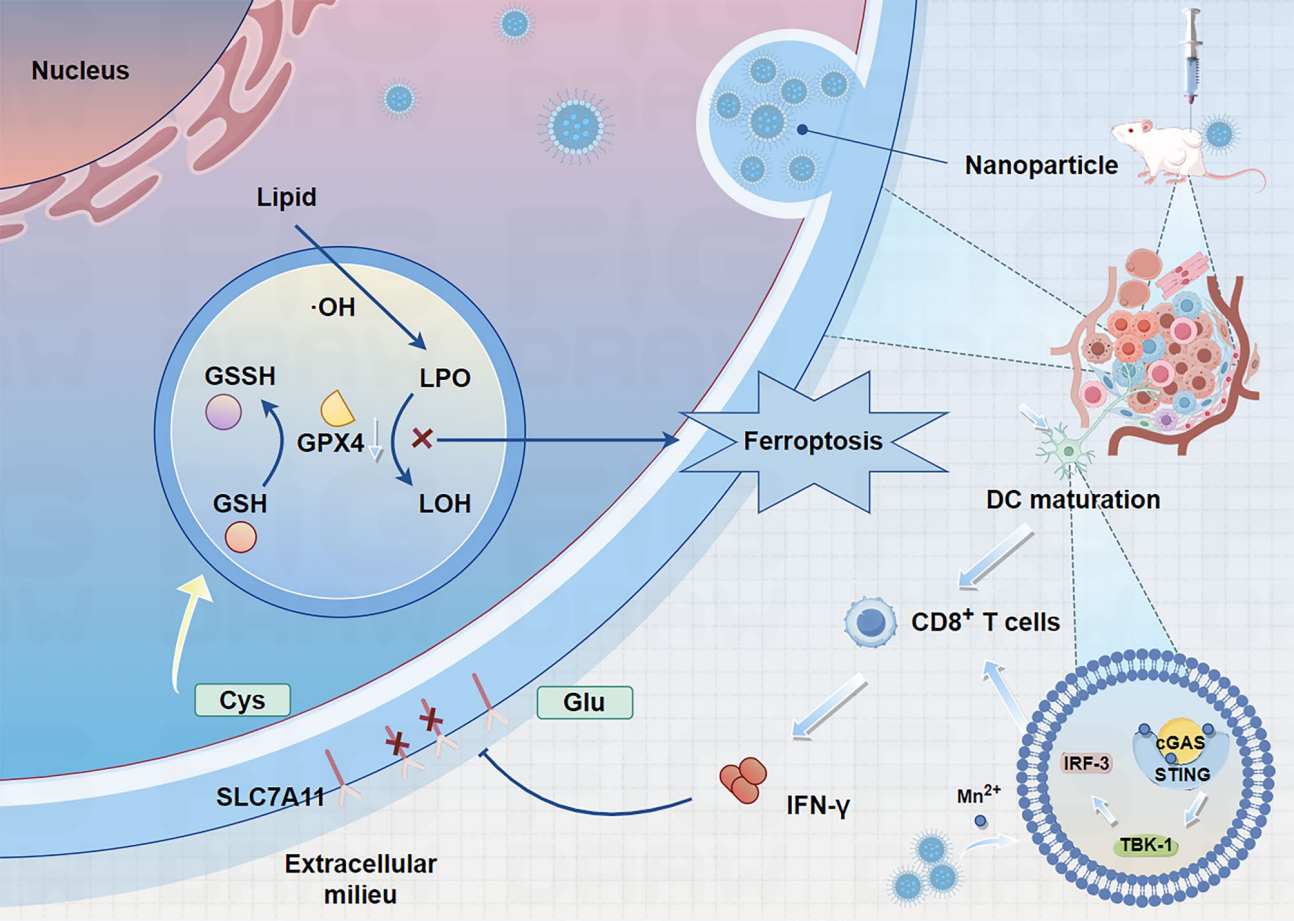
Schematic diagram of the synthesis method and anti-tumor mechanism of MGNH

Wang et al. developed an ultra-small Janus-structured MnFe₂O₄@NaGdF₄@ NLG919@HA(MGNH) nanocomplex engineered to overcome the immunosuppressive TME through synergistic ferroptosis activation and immune modulation (Fig. 26) [161]. The hierarchical design integrates tumor-targeting HA with a heterostructured MnFe₂O₄@NaGdF₄ (MG) core and immune checkpoint inhibitor NLG919, enabling triple-modal theranostic functionality. HA-mediated active targeting ensures precise tumor accumulation of both the MG Janus nanoparticles and NLG919, while the MG core serves as a dual T₁/T₂ MRI contrast agent and photothermal converter for imaging-guided photothermal therapy (PTT) [161]. The nanocomplex orchestrates ferroptosis through three-pronged catalysis, which depletes GSH, amplifies ROS production through H_2_O_2_ conversion, and suppresses GPX4 expression to drive lethal LPO accumulation [161]. Concurrently, Mn^2+^ ions activate the cGAS-STING pathway to stimulate antitumor immunity, synergizing with NLG919’s blockade of the IDO-mediated tryptophan/kynurenine axis to inhibit Treg differentiation and reverse immunosuppression [161]. The ultra-small Janus architecture enhances tumor penetration and renal clearance and mitigates long-term biosafety concerns [161]. By unifying ferroptosis potentiation (through chemodynamic therapy/PTT), immune reprogramming, and real-time imaging within a single biodegradable platform, this work establishes a paradigm for next-generation nanomedicines that concurrently dismantle biochemical and immunological barriers in solid tumors, offering a clinically translatable strategy for precision TME remodeling [161].

**Fig. 27:**
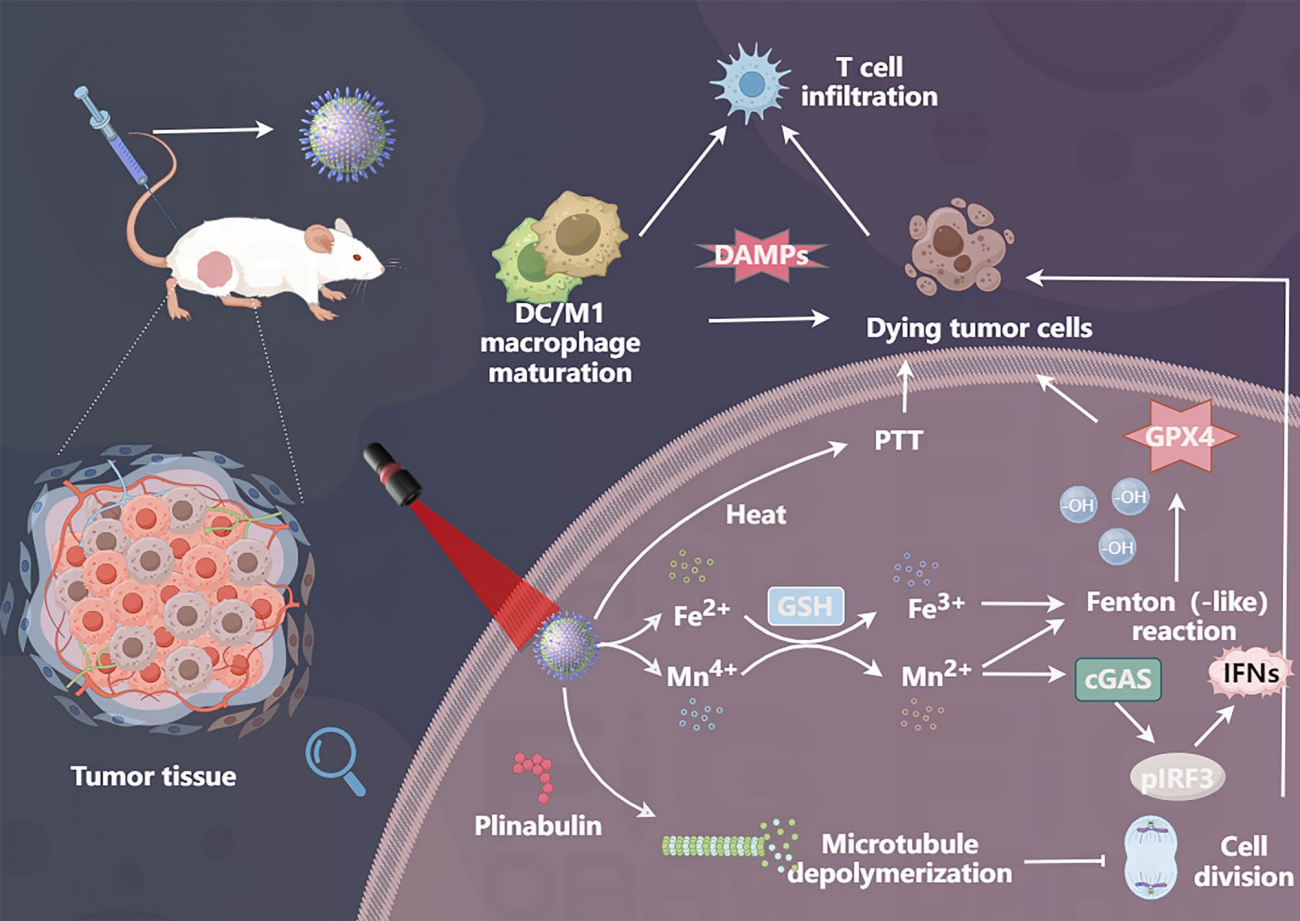
Schematic illustration of FMPH preparation and molecular mechanisms for effective tumor therapy

Li et al. developed an engineered Fe-MOF@MnO₂@PB@HA (FMPH), a multifunctional nanoplatform integrating an iron-based MOF (Fe-MOF) core encapsulating plinabulin (PB), a microtubule-destabilizing and vascular-disrupting agent that inhibits tumor proliferation, with a manganese dioxide (MnO₂) shell enhancing chemodynamic therapy (CDT) through GSH depletion and •OH generation and HA surface modification for tumor-targeted delivery and systemic toxicity reduction (Fig. 27) [161]. The MnO₂ component further enables near-infrared (NIR) -responsive hyperthermia while synergistically amplifying cGAS-STING activation to induce ICD and antitumor immune activation. In vitro and in vivo studies have demonstrated robust tumor suppression by FMPH, with NIR-triggered photothermal therapy augmenting the therapeutic efficacy. This multimodal nanomedicine, which combines ferroptosis induction, immunomodulation, targeted drug delivery, and photothermal enhancement, represents a promising strategy for synergistic cancer therapy [161].

This section demonstrates that rationally designed nanomedicines (e.g., PMZFNs, IFN-γ/uMn-LDHs, CMArg@Lip, FeMn@cGAMP@M) can simultaneously activate ferroptosis and cGAS-STING signaling across diverse cancers through synergistic mechanisms: (1) metal ion-mediated catalysis (Fe²⁺/Mn²⁺) depletes antioxidants (GSH/GPX4), driving LPO and ferroptosis; (2) ferroptosis-derived DAMPs (mtDNA, HMGB1) or co-delivered STING agonists (cGAMP, diABZi) activate cGAS-STING, amplifying ICD and dendritic cell/T cell responses; (3) self-reinforcing cycles emerge when STING-driven IFN-γ suppresses SLC7A11 to enhance ferroptosis, while ferroptosis liberates antigens/DAMPs to potentiate STING immunity. These platforms can overcome therapeutic barriers (drug resistance and immunosuppression) through tumor-targeted delivery, TME-responsive activation, and combinatorial remodeling of the tumor microenvironment, thereby achieving potent antitumor efficacy with minimal toxicity in preclinical models.

Critical unresolved challenges include elucidating the long-term biosafety profiles of metal-ion-releasing nanomaterials (e.g., Mn²⁺/Fe³⁺ accumulation), defining biomarkers to predict and monitor the dynamic interplay between nanomedicine -induced ferroptosis and immune activation in clinical settings, and addressing tumor heterogeneity-driven variations in therapeutic response across cancer types. Future studies must prioritize clinical translation by optimizing scalable nanomanufacturing, establishing tissue-specific biodistribution and clearance mechanisms, developing imaging-guided strategies to track the spatiotemporal coactivation of both pathways in vivo, and designing adaptive “logic-gated” nanosystems that can intelligently balance the ferroptotic and immunostimulatory outputs on the basis of real-time TME cues to prevent systemic hyperinflammation or off-target toxicity.

## Conclusions and perspectives

Targeting the cGAS-STING pathway in tumor immunotherapy has recently emerged as an important research direction, promoting the development of small-molecule STING agonists. Due to the limitations of STING agonists, including adverse effects, nonspecific accumulation, and rapid clearance, extensive research on nanomaterials has emerged as a breakthrough for safe and effective cancer immunotherapy with STING agonists. A growing body of evidence has shown that cGAS-STING interacts with ferroptosis in cancer, which can be leveraged by nanomedicines to offer a novel cancer regimen. Therefore, we reviewed the role of the interplay between cGAS/STING and ferroptosis in cancer genesis. We then focused on providing an overview of the latest findings and emerging concepts that leverage the interplay between cGAS-STING and ferroptosis by nanomedicine to kill cancers.

Although preliminary progress has been made in the use of nanomedicines targeting the interplay between cGAS-STING and ferroptosis, studies on nanomedicines that can simultaneous target cGAS-STING and ferroptosis in tumors are still in their infancy. Several critical challenges remain to be addressed in future studies. First, the mechanisms underlying the interaction between cGAS-STING and ferroptosis in tumors remain unclear and warrant further investigation. Such research could lay a solid foundation for precise targeting of the interplay between cGAS-STING and ferroptosis, thereby advancing therapeutic strategies. Future research should aim to design and develop stimuli-responsive nanomaterials (e.g., pH-/ROS-/enzyme-activated systems) to co-deliver cGAS-STING agonists (e.g., CDNs) and ferroptosis inducers (e.g., FINs or iron-loaded nanoparticles) with spatiotemporal precision. Multi-omics approaches and CRISPR screening should be used to decode the molecular interplay between cGAS-STING signaling and ferroptosis execution in tumor and immune cells, and immunocompetent tumor organoids or humanized mouse models should be employed to evaluate dual-target nanomedicine efficacy, immune memory, and systemic safety. AI-driven nanoparticle design and molecular dynamics simulations can be utilized to optimize the dual-targeting efficiency and pharmacokinetics.

Future research directions remain an open conundrum, and the mechanisms underlying the crosstalk between cGAS-STING and ferroptosis remain to be determined. Future studies should aim to investigate how cGAS-STING activation regulates ferroptosis sensitivity (e.g., via IFN-driven lipid metabolism reprogramming) and vice versa. Hybrid nanomaterials (e.g., metal-organic frameworks or lipid-polymer composites) should be designed to synchronously target tumor cells (ferroptosis) and immune cells (cGAS-STING activation). A central issue that has not been addressed is the screening of translational biomarkers, i.e., identifying predictive biomarkers (e.g., STING /GPX4 expression ratios and LPO levels) to stratify patients for dual-targeted nanotherapy. The development of engineered tumor-selective targeting ligands (e.g., anti-PDL1-decorated nanoparticles) to minimize off-target effects on healthy tissues is also a key focus.

In summary, emerging preclinical studies have demonstrated the feasibility of combining cGAS-STING activation with ferroptosis induction using nanomaterials, leveraging the unique properties of this approach, such as tumor-specific drug delivery, controlled release, and synergistic immunomodulatory effects. However, challenges persist, including an insufficient mechanistic understanding of cGAS-STING-ferroptosis crosstalk, suboptimal spatiotemporal control of dual-target nanocarriers, and potential off-target toxicity. Bridging the gap between mechanistic complexity and nanomedicine innovations is critical for unlocking the full potential of dual cGAS-STING/ferroptosis targeting for next-generation cancer therapies.

## Supplementary Information

Below is the link to the electronic supplementary material.

Supplementary Material 1

## Data Availability

No datasets were generated or analysed during the current study.
