# Supplementary material for: Targeting the interplay of cGAS-STING and ferroptosis by nanomedicine in the treatment of cancer
Source: J Exp Clin Cancer Res. 2025 Aug 22;44:249. doi: 10.1186/s13046-025-03520-6 (PMC12372235; doi:10.1186/s13046-025-03520-6)
Supplement: Supplementary file 1 — Supplementary Material 1 [file 13046_2025_3520_MOESM1_ESM.pdf]

CERTIFICATE OF ENGLISH EDITING

This document certifies that the paper listed below has been edited to ensure that the language is clear and free of errors. The logical presentation of ideas and the structure of the paper were also checked during the editing process. The edit was performed by professional editors at Editage, a division of Cactus Communications, in cooperation with Lippincott. The intent of the author's message was not altered in any way during the editing process. The quality of the edit has been guaranteed, with the assumption that our suggested changes have been accepted and have not been further altered without the knowledge of our editors.

Title

Targeting the interplay of cGAS-STING and ferroptosis by nanomedicine in the treatment of cancer

Authors

Chunfei Li, Wenyan Zhao, Donghua Geng, Yuzi Jin, Wenzheng Guan

Order No.

UGDNX_20

Lippincott®

Signature

Prabh Grewal,
Senior Vice President,
Editage

Date of Issue
August 05, 2025
